# Low-dose, non-supervised, health insurance initiated exercise for the treatment and prevention of chronic low back pain in employees. Results from a randomized controlled trial

**DOI:** 10.1371/journal.pone.0178585

**Published:** 2017-06-29

**Authors:** Sven Haufe, Klaus Wiechmann, Lothar Stein, Momme Kück, Andrea Smith, Stefan Meineke, Yvonne Zirkelbach, Samuel Rodriguez Duarte, Michael Drupp, Uwe Tegtbur

**Affiliations:** 1 Institute of Sports Medicine, Hannover Medical School, Hannover, Germany; 2 Institute of Clinical Pharmacology, Hannover Medical School, Hannover, Germany; 3 Institute of Biometry, Hannover Medical School, Hannover, Germany; 4 AOK health insurance, Hannover, Germany; Universite de Nantes, FRANCE

## Abstract

**Objective:**

Back pain is a major problem requiring pragmatic interventions, low in costs for health care providers and feasible for individuals to perform. Our objective was to test the effectiveness of a low-dose 5-month exercise intervention with small personnel investment on low back strength and self-perceived pain.

**Methods:**

Two hundred twenty-six employees (age: 42.7±10.2 years) from three mid-size companies were randomized to 5-month non-supervised training at home (3 times/week for 20 minutes) or wait-list-control. Health insurance professionals instructed the participants on trunk exercises at the start and then supervised participants once a month.

**Results:**

Muscle strength for back extension increased after the 5-month intervention with a significant between-group difference (mean 27.4 Newton [95%CI 2.2; 60.3]) favoring the exercise group (p = 0.035). Low back pain was reduced more in subjects after exercise than control (mean difference –0.74 cm [95%CI –1.17; –0.27], p = 0.002). No between-group differences were observed for back pain related disability and work ability. After stratified analysis only subjects with preexisting chronic low back pain showed a between-group difference (exercise versus controls) after the intervention in their strength for back extension (mean 55.7 Newton [95%CI 2.8; 108.5], p = 0.039), self-perceived pain (mean –1.42 cm [95%CI –2.32; –0.51], p = 0.003) and work ability (mean 2.1 points [95%CI 0.2; 4.0], p = 0.032). Significant between-group differences were not observed in subjects without low back pain: strength for back extension (mean 23.4 Newton [95%CI –11.2; 58.1], p = 0.184), self-perceived pain (mean –0.48 cm [95%CI –0.99; 0.04], p = 0.067) and work ability (mean –0.1 points [95%CI –0.9; 0.9], p = 0.999). An interaction between low back pain subgroups and the study intervention (exercise versus control) was exclusively observed for the work ability index (p = 0.016).

**Conclusion:**

In middle-aged employees a low-dose, non-supervised exercise program implemented over 20 weeks improved trunk muscle strength and low back pain, and in those with preexisting chronic low back pain improved work ability.

## Introduction

Non-specific lumbar pain is a major contributor to sick days in older employees, but it also affects younger workers [1]. The consequences are restricted health but also lower productivity and the possibility of not being able to work. Direct costs for low back pain (LBP) are estimated between $20 billion and $98 billion in the US; with indirect annual costs estimates as high as $200 billion [2, 3], underlining non-specific LBP as a major socio-economic problem in industrialized countries.

Exercise training is a commonly used therapy for the conservative treatment of LBP [4, 5]. Exercise interventions in studies are often supervised, with individual coaching and high-dose in exercise volume and/or intensity [6] which makes it difficult to simply extrapolate the results and concepts to exercise therapy in daily medical routine. Inconsistent results have been reported from interventions on low-dose exercise programs for back pain prevention and management [7–12]. Only a few studies were conducted in collaboration with public or institutional health insurance companies to gather information about people who had been sick-listed [7, 13] or data for retrospective analyses [14, 15]. Most of these trials were designed and guided by academic investigators who provided feedback and motivation to study participants. Therefore, transferability to a real-life setting where prevention and rehabilitation programs offered by the public health care are restricted in personnel and financial means is difficult. At the moment, data from prospective-controlled trials testing the effectiveness of exercise interventions that are independently planned and conducted by health insurances companies are very limited [16].

As a consequence we conducted a prospective study to evaluate the effects of a recently implemented program from a large health insurance company in Germany designed to provide a pragmatic exercise intervention in terms of personnel and financial demands. We hypothesized that this non-supervised and low-dose exercise intervention for the trunk muscles will improve the lower back strength, back pain and work ability of employees with and without existing chronic LBP.

## Subjects and methods

### Subjects

To recruit volunteers we organized local informative events for employees and distributed advertisements via email and the internal intranet in three medium-sized companies (company 1: overall employees’ n = 460 [49% desk work, 51% manufacturing]; company 2: overall employees’ n = 391 [100% desk work]; company 3: overall employees’ n = 1342 [46% desk work, 54% manufacturing]. Other than receiving individual instruction for exercises no additional incentives were offered by the health insurance or the employer. According to pre-study defined inclusion and exclusion criteria, which were tested after recruitment we included female and male employees between 18 and 67 years. Exclusion criteria were acute or chronic infections and any diseases that preclude realization of an exercise intervention. Pregnant or breast feeding women were also excluded.

### Ethical approval

This study was carried out in accordance with the Declaration of Helsinki and current guidelines of good clinical practice. The ethics committee of Hannover Medical School approved the study (EC number: 6565) and written informed consent was obtained before study entry.

### Study design

This was a prospective, randomized, parallel-group, and single-blind (assessor blind) study conducted between January and November 2014. The intervention was performed as a cooperation project between the AOK health insurance and Hannover Medical School (ClinicalTrials.gov Identifier: NCT02029131). Study design, statistical planning and analysis, ethical approval, and inclusion of volunteers, as well as data preparation and manuscript writing were in the responsibility of the Institute of Sports Medicine in cooperation with the Institute of Biometry at Hannover Medical School. Strength testing, exercise prescription and counselling during the intervention were done by qualified personnel of the AOK. Volunteers were centrally randomized 1:1 into an intervention- and a waiting-control group by using a computer-generated list of random numbers stratified for the company and presence of chronic low back pain. For randomization, blocks were used to ensure that the groups were of equal size. Block length was varied as a safeguard so allocation would not be predictable for the persons responsible for the randomization. The primary outcome measurement was isometric back extension strength which is an objective measure associated with low back pain, disability and function [17–19]. Study personnel assessing the primary endpoint at baseline and after 5 months were blinded for the randomized group allocation of subjects.

### Anthropometric and strength assessments

We determined body weight and height, and assessed fat free mass (FFM) and fat mass by segmental, multi-frequency bio impedance analysis (InBody 720, Biospace, Seoul, Korea). By introducing an electrical current throughout the body the estimated total body water is used to calculate FFM. The fat mass is calculated by subtracting FFM from body weight. Furthermore we asked subjects for their self-rated intensity of LBP during the last seven days on a 10 centimeters visual analog scale (VAS). In patients with chronic LBP a change of 1.5 cm on the VAS is considered as a minimally important change [20]. We handed out questionnaires for the estimation of low back pain related disability (Oswestry disability index) [21], health-related quality of life (short form 36 [SF-36]) [22], daily physical activity (Freiburger Activity questionnaire) [23], and work ability (work ability index [WAI]) [24]. The Oswestry questionnaire is a self-administered questionnaire including 10 items to assess the extent of back pain and difficulties in carrying out nine different daily activities, such as lifting, walking and sitting. Each item is scored from 0 to 5, and higher values represent greater disability. The total score is usually expressed as a percentage [25] for which an improvement of 10% is considered as important [20]. The SF-36 questionnaire measures the health-related quality of life with 8 subscales and two sum scales related to mental and physical aspects. For all scales, a score of 0 points means a minimum and a score of 100 points a maximum quality of life. The Freiburger activity questionnaire is a reliable and valid tool for determining the health-effective physical activity of adults [23]. The results of this questionnaire are the total score and the sport score, both of which are specified as metabolic equivalents of task (MET)-hours per week. The WAI questionnaire (short form) contains 7 questions concerning work, work ability and health, resulting in a total score ranging from 7 to 49, with higher values representing greater work ability. We also asked for the presence of chronic low back pain (yes or no) using a definition from the “Robert Koch Institute, Berlin, Germany” which was specified as having experienced low back pain almost every day for a minimum of three months per year. Finally all subjects underwent strength testing for the trunk with the Back-check® 607 apparatus (Dr. WOLFF® Sports & Prevention GmbH, Arnsberg; Germany) a mobile device to assess isometric strength [26]. After explaining the testing procedure we positioned subjects in the testing system in in an upright standing position (0° hip extension). They were asked to perform one pretest without maximum force to simulate the test movement. Subjects were then advised to press once, with a maximum voluntary force against a fixed pad that was individually adjusted to the middle of the shoulder blade for 10 seconds and the highest force during the 10-second test was taken for analysis. Similarly, subjects had to press once against a pad adjusted to the left or right shoulder (same starting position) to assess isometric strength for lateral flexion to both sides.

### Study intervention

After randomization individual counselling by one of two physiotherapist of the AOK health insurance took place once at baseline and once every month during the 5-month intervention only for subjects allocated to the exercise group. Exercise planning and guidance was entirely the responsibility of the AOK physiotherapist. The training was designed as a 20-minute non-supervised exercise session three times per week, which was performed at home or during regular pauses at work. At the first meeting, subjects received comprehensive instructions and demonstrations of four to six exercises chosen from a list (see S1 Dataset for the list of exercises; illustrations from PhysioWorkout, PhysioNetzwerk GmbH, Delmenhorst, Germany). They also received a printed manual with illustrations of selected exercises and their individual exercise prescription. Exercises were possible to conduct without additional equipment provided by the physiotherapist or acquired by the subjects. Training included generally established exercises for the trunk musculature particularly for the lower back including exercises for extensor muscles of the lower spine, abdominal muscles, muscles involved in lateral trunk flexion and muscles of the rotator cuff of the shoulder. Practicing was performed with a moderate contraction velocity, with concentric, eccentric or isometric muscle actions with specified repetitions in 2 or 3 sets. It mostly involved multiple joint exercises with many muscle groups [27]. During the regular counsellings the physiotherapist supervised and corrected the exercise performance, adapted the exercises in regard to the sets, repetitions and intensity of training at its own discretion, and addressed individual questions. Subjects in the waiting-control group were asked to continue their current lifestyle and offered the opportunity to undergo the same intervention after finishing the study.

### Sample size and statistical analysis

Based on a previous randomized intervention study by Moon HJ et al. [28] we calculated that we had to include at least 200 volunteers to observe a significant increase in the isometric strength for back extension. Sample size calculation was performed in nQuery Advisor 7.0 with a two-group t-test, a type-I-error of 5% and a power of 80%.

The statistical analysis was based on the intention-to-treat principle, including all randomized subjects. Missing values were replaced by the baseline observation carried forward method. Data were first tested for normality of distribution with the Kolmogorov-Smirnov test. Beside the primary outcome back extension strength, secondary outcomes included lateral trunk strength, perceived LBP, LBP related disability, work ability, daily physical activity, and body composition. For all outcomes an Analysis of Covariance model (ANCOVA) was used with a mean change from baseline of isometric strength for back extension (20 weeks-baseline) as a response variable. Explanatory variables were the stratification factors (company and chronic back pain at baseline), the parameter at baseline and group (exercise- vs. control). For descriptive analysis absolute frequencies were calculated for categorical variables. For continuous variables the mean and standard deviation (SD) were used in text and tables and the mean and standard error (SE) were used in figures. To test for within-group differences from baseline to end of intervention, a two-sided t-test for paired samples was used. Univariate associations between parameters were tested using *Pearson’s* correlation coefficient. For the analysis of interactions between subgroups stratified for the presence of low back pain at baseline [LBP or no LBP] and the study intervention [exercise or control] an interaction term (subgroup x study intervention) was included in the primary ANCOVA model. The type-I-error was set to 5% (two-sided). All statistical analyses were performed with IBM SPSS 22 (IBM Corporation, NY, US).

## Results

**Fig 1:**
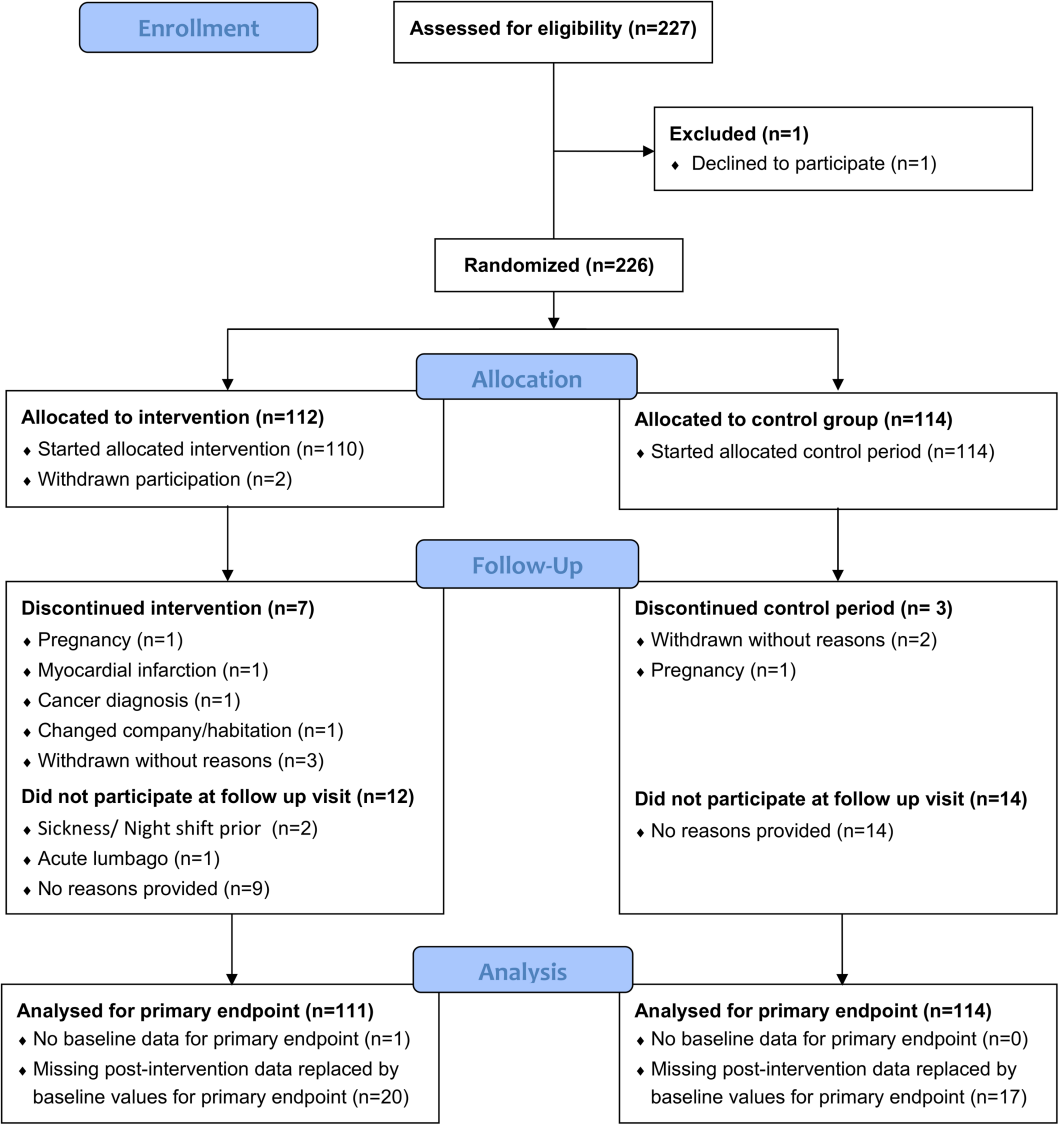
CONSORT flow diagram of the progress throughout the study.

Of the approximately 800 employees who received the study information 227 were interested in participating and were invited to be screened for eligibility. Two-hundred twenty-six subjects were randomized (absolute numbers and percentage relative to overall employees in the companies: company 1: n = 36 (8%), company 2; n = 26 (7%), company 3: n = 164 (12%). One-hundred eighty-eight (83%) completed the intervention phase (for details see Fig 1).

Of those, muscle strength for back extension was not measured at baseline in one subject. For the secondary outcomes baseline data could not be assessed for the following: Oswestry Disability Index (n = 9), SF-36 (n = 14), daily physical activity (n = 9), WAI (n = 10), and fat- and fat-free mass (n = 5). The study groups did not significantly differ at baseline for sex, age, body weight, body composition, blood pressure, daily physical activity, work ability, low back pain, and back pain related disability (Table 1). There was no report of any adverse events during the study period.

**Table 1:** Subject characteristics at baseline.

	Exercise group	Control group	p-value
**n (women/ men)**	112 (48/64)	114 (43/71)	0.43
**Age (years)**	43.5 ± 9.7	41.9 ± 10.6	0.24
**Body weight (kg)**	82.3 ± 17.2	84.1 ± 14.9	0.39
**Body mass index (kg/m**^**2**^**)**	26.7 ± 4.5	26.9 ± 4.0	0.81
**Body fat (%)**	27.2 ± 9.2	27.0 ± 9.1	0.88
**Fat free mass (kg)**	59.1 ± 14.4	61.1 ± 12.2	0.27
**Systolic blood pressure (mm/Hg)**	134 ± 14	134 ± 14	0.99
**Diastolic blood pressure (mm/Hg)**	82 ± 8	81 ± 9	0.90
**Chronic low back pain (yes/no)**	28/84	27/87	0.82
**Daily physical activity (MET-hours/wk)**	27.4 ± 20.9	30.0 ± 28.3	0.22
**Work ability index (total points)**	39.4 ± 5.2	38.9 ± 5.7	0.53

MET = metabolic equivalent of task; no significant differences were observed between groups as analyzed with Students T-Test for unpaired samples or the chi-square test, data are mean ± SD.

### Effects of the intervention on primary and secondary outcomes

**Fig 2:**
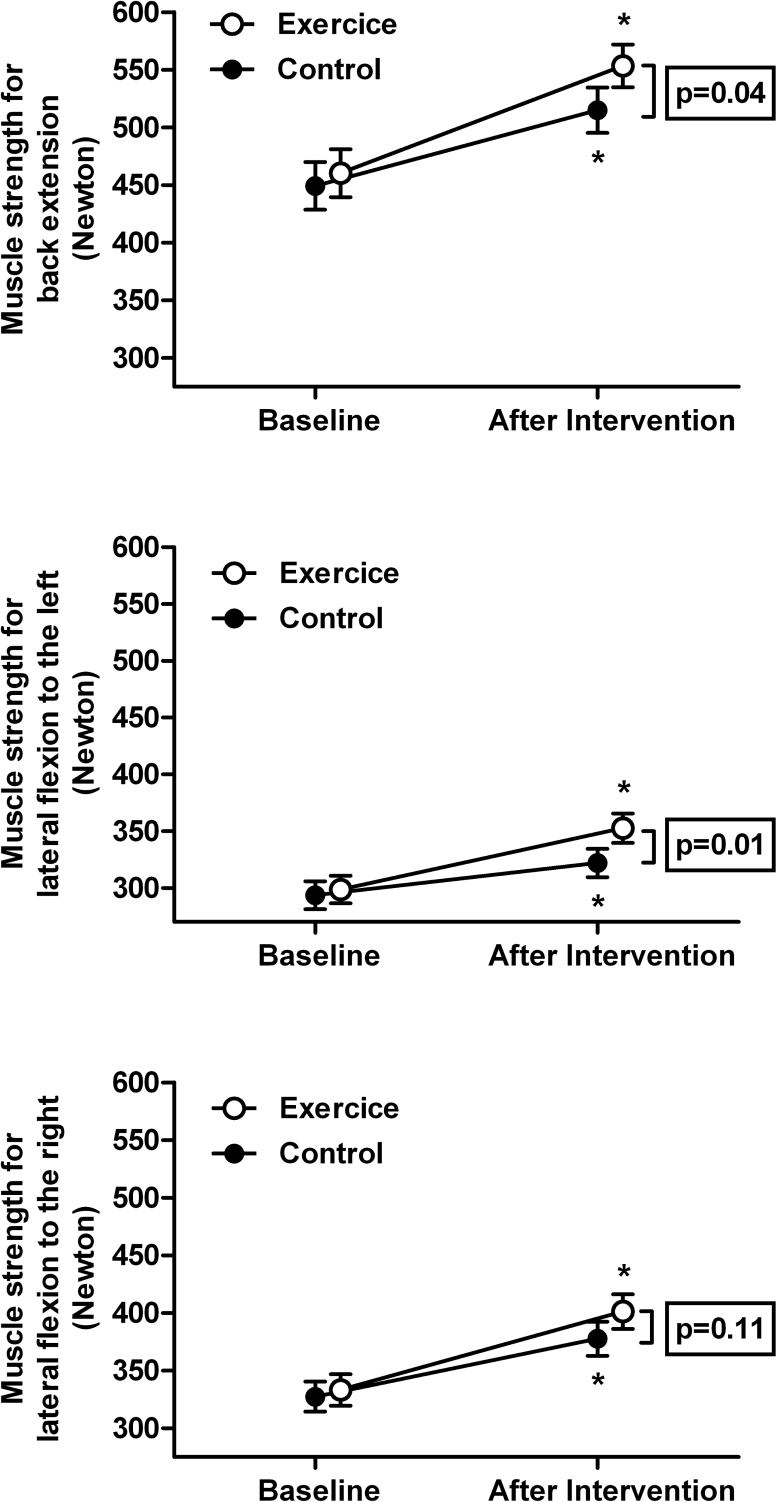
Maximum voluntary force before and after 5 months of exercise. Maximum force at baseline and after the 5-month intervention for isometric low back extension (upper panel), lateral flexion to the left side (middle panel) and lateral flexion to the right side (lower panel) for the exercise and control group. Data are mean ± SE. * indicates p<0.05 for within-group differences (pre versus post-intervention) as analyzed with Students t-test for paired samples. The framed p-value represents the between-group differences (exercise- versus control group) over time as analyzed with an ANCOVA model.

Isometric strength for low back extension after the 20-week intervention increased significantly more in the exercise group compared to the control group (Table 2 and; Fig 2A).

**Table 2:** Changes from baseline for each study group and mean differences between groups for primary and key secondary outcomes.

	Exercise group	Control group	Mean differences and CI between groups	p-value* between groups
**Strength for back extension (N)**	93.1 ± 125.4	65.7 ± 120.6	27.4 [2.2; 60.3]	0.035
**Strength for trunk flexion to the left (N)**	54.1 ± 79.1	28.5 ± 78.6	25.6 [7.1; 46.9]	0.008
**Strength for trunk flexion to the right (N)**	68.0 ± 92.7	50.3 ± 85.9	17.8 [–4.1; 42.1]	0.107
**Low back pain on the VAS (cm)**	–0.90 ± 2.26	–0.17 ± 1.84	–0.74 [–1.17; –0.27]	0.002
**Oswestry disability score (%)**	–1.32 ± 4.72	–0.58 ± 4.49	–0.75 [–2.05; 0.30]	0.142
**Work ability index (total points)**	1.08 ± 3.13	0.71 ± 3.53	0.36 [–0.32; 1.32]	0.228

VAS = visual analog scale

*results adjusted for baseline, company and chronic low back pain; data are mean ± SD, or mean [95% CI] for differences between groups.

The increase for lateral flexion strength to the left was significantly higher for the exercise group over time compared to controls (Table 2, Fig 2B). There was no difference between study groups for lateral flexion strength to the right side (Table 2). See S1 Table for the full estimation results.

**Fig 3:**
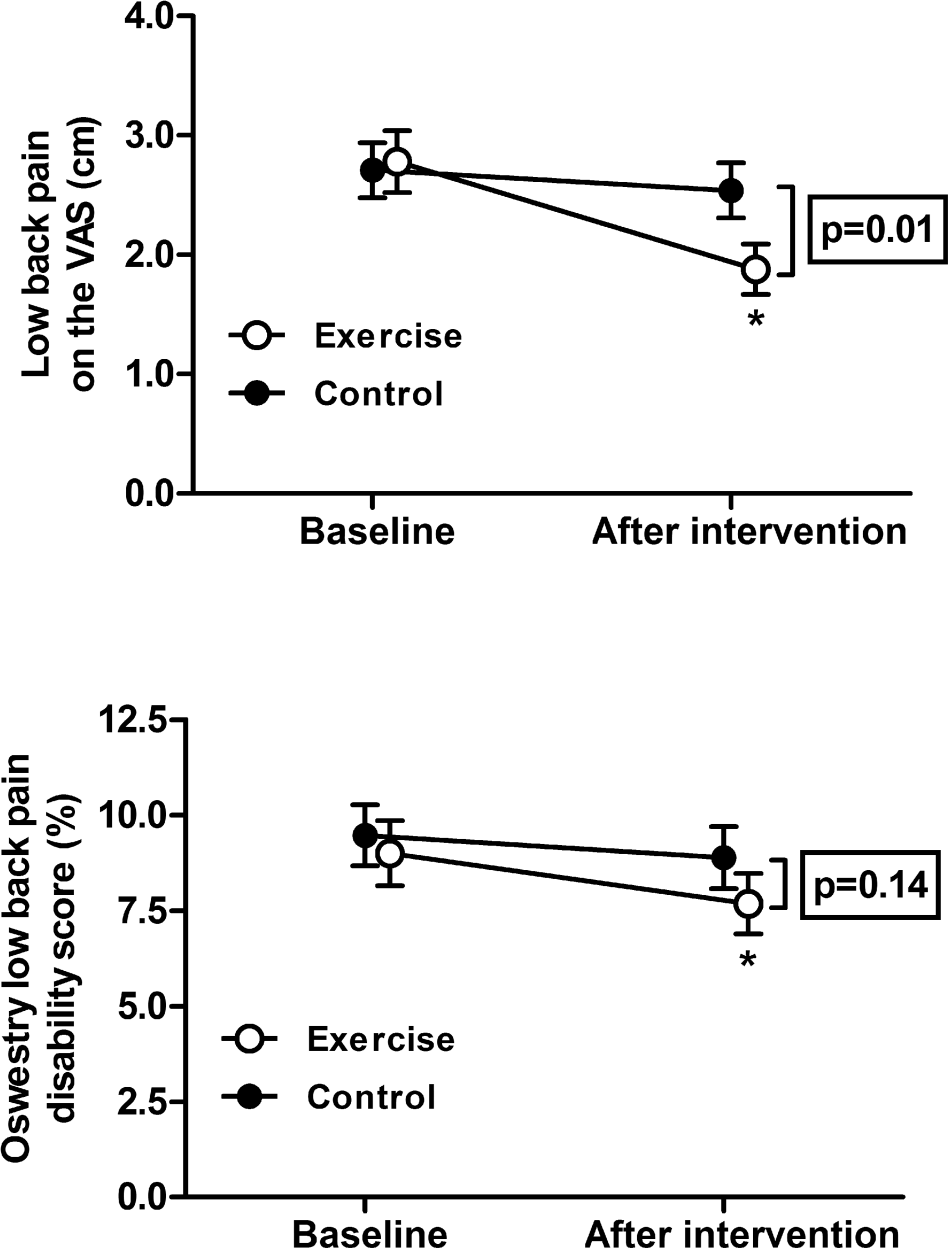
Low back pain and related disability before and after 5 months of exercise. Low back pain as assessed with a 10-cm visual analog scale (VAS, upper panel) and the low back pain disability score from the Oswestry Low Back Pain Disability Questionnaire for the exercise and control group at baseline and after 5 months intervention. Data are mean ± SE. * indicates p<0.01 for within-group differences (pre versus post-intervention) as analyzed with Students t-test for paired samples. The framed p-value represents between-group differences (exercise- versus control group) over time as analyzed with an ANCOVA model.

The changes in LBP as assessed via the VAS were significantly greater for the exercise group compared to the control group (Table 2, Fig 3). For the Oswestry Disability Index there were no significant differences between groups (Table 2, Fig 3).

There was no difference between groups over time for the work ability index (Table 2). Daily physical activity was higher for subjects in both groups at the end of the 20 the week intervention with no differences between groups (p = 0.76). No differences were observed between study groups for changes in health-related quality of life over time for neither sub-scale of the SF-36.

Changes in body weight and BMI from baseline were not different between groups (p = 0.42 for body weight; p = 0.62 for BMI). The percentage body fat decreased in both groups with no significant changes for fat free mass (for body fat p = 0.82 between groups, and for fat free mass: p = 0.91 between groups).

### Differential response of training for individuals with and without chronic low back pain

**Fig 4:**
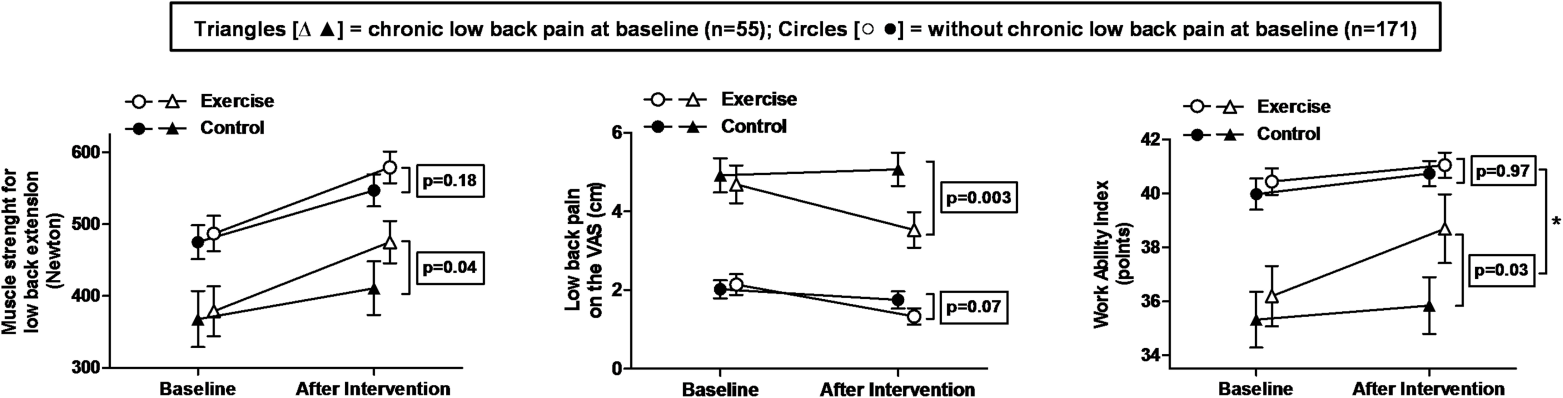
Effects of 5 months of exercise for subjects with or without pre-existing chronic low back pain. Maximum force for isometric low back extension (left panel), low back pain as assessed with a 10 cm visual analog scale (VAS, middle panel), and the work ability index (total score) from the work ability questionnaire (right panel) before and after 5 months exercise or control. Subjects are stratified for the presence of chronic low back pain at baseline, defined as having experienced low back pain almost every day for a minimum of three months per year. Data are mean ± SE. The framed p-values are given for between-group differences (exercise- versus control group) over time as analyzed with an ANCOVA model. * indicates p<0.05 for the interaction (subgroup [chronic LBP or no chronic LBP] x intervention [exercise or control]) included as covariate in the primary analysis model.

After performing a subgroup analysis based on stratification for preexisting chronic LBP we observed at baseline similar body weight, BMI, and daily physical activity but higher age (45.4±8.5 vs. 41.8±10.6 ys.; p<0.05), percentage body fat (30.1±8.8 vs. 26.2±9.1%), low back pain (4.8±2.4 vs. 2.1±2.3 cm) back pain related disability (17.1±9.5 vs. 6.8±6.6%), and percentage of women (56% vs. 35%) for subjects with preexisting chronic LBP (for all: p<0.01). In contrast the work ability (35.8±5.6 vs. 40.2±5.0 points), and the isometric strength for low back extension (373±191 vs. 480±222 N) was lower for subjects with LBP than for those without chronic LBP (for all: p<0.01). After the 5-month program strength for low back extension increased significantly after exercise only in subjects reporting chronic LBP at baseline (Fig 4). Increases in muscle strength with lateral flexion to the left, work ability, as well as decreases in the LBP intensity were also exclusively observed in subjects with preexisting chronic LBP (Table 3, Fig 4 and S2 Table). A significant interaction (LBP subgroup x study intervention) was only observed for the work ability index (interaction: p = 0.016). When including age or gender as potential confounding factors in the primary analysis model, both were not significant confounders for any analyzed outcome.

**Table 3:** Baseline data and differences between groups over time for subjects with and without chronic low.

	Subjects without chronic low back pain				Subjects reporting chronic low back pain			
	Exercise baseline	Controls baseline	Mean differences and CI between groups over time	p-value for between-group differences	Exercise baseline	Controls baseline	Mean differences and CI between groups over time	p-value for between-group differences
n (women/ men)	84 (32/52)	87 (28/59)			28 (16/12)	27 (15/12)		
Age (years)	43.5 ± 10.4	41.1 ± 10.7			46.5 ± 6.5	44.4 ± 10.2		
Body weight (kg)	81.8 ± 15.8	84.8 ± 15.0	–0.28 [–1.17; 0.61]	0.536	83.7 ± 20.9	81.8 ± 14.7	–0.58 [–2.22; 1.08]	0.487
Body mass index (kg/m^2^)	26.4 ± 4.0	26.9 ± 4.1	0.10 [–0.26; 0.47]	0.583	27.8 ± 5.6	26.9 ± 3.7	–0.01 [–0.57; 0.56]	0.986
Body fat (%)	26.2 ± 9.3	26.2 ± 8.9	–0.32 [–1.03; 0.38]	0.495	30.4 ± 8.3	29.8 ± 9.6	0.71 [–0.60; 2.01]	0.284
Fat free mass (kg)	59.9 ± 14.9	62.2 ± 11.9	0.11 [–0.36; 0.58]	0.489	56.7 ± 12.8	57.4 ± 12.6	–0.50 [–1.57; 0.58]	0.357
Isometric strength for trunk flexion to the left (N)	311 ± 125	305 ± 130	20.2 [–3.3; 43.5]	0.092	260 ± 129	257 ± 128	48.8 [10.8; 86.7]	0.013
Isometric strength for trunk flexion to the right (N)	341 ± 144	334 ± 134	13.9 [–14.3; 42.2]	0.331	309 ±143	304 ± 154	34.9 [–3.1; 72.9]	0.071
Daily physical activity (MET-hours/wk)	27.6 ± 19.5	32.2 ± 34.4	–0.89 [–9.29; 7.51]	0.835	25.7 ± 19.8	29.3 ± 22.3	–1.41 [–11.05; 8.23]	0.770
Oswestry disability score (%)	6.7 ± 6.7	6.9 ± 6.5	–0.40 [–1.56; 0.75]	0.494	16.0 ± 10.50	18.3 ± 8.3	–2.58 [–5.95; 0.79]	0.131
SF 36 physical score (points)	52.9 ± 6.3	51.6 ± 7.3	0.17 [–1.48; 1.82]	0.838	45.7 ± 8.5	41.0 ± 8.1	–0.01 [–3.42; 3.40]	0.996
SF-36 mental score (points)	48.8 ± 7.3	48.0 ± 9.4	0.59 [–1.25; 2.42]	0.531	43.5 ± 11.5	49.2 ± 8.7	2.67 [–1.72; 7.05]	0.227

MET = metabolic equivalent of task, SF-36 = short form 36, Differences between intervention groups were analysed by an analysis of covariance model with the mean difference (baseline to follow-up) of the respective parameter as response variable and the baseline value of the parameter, company and intervention group (exercise versus control) as explanatory variables. No significant interaction (subgroup [low back pain or no low back pain] x intervention [exercise or control]) was detected for any parameter. Data are mean ± SD or mean [95% CI] for differences between groups over time.

## Discussion

Our main finding is that a 5-month pragmatic exercise intervention that was designed and conducted by a large health insurance company improved the trunk muscle strength and perceived low back pain in middle-aged employees. After stratification on preexisting chronic LBP exercise training improved strength for back extension and lateral flexion to the left, work ability and low back pain only within the subgroup of subjects presenting with LBP. However, a significant interaction between LBP subgroups and the study intervention was exclusively observed for the work ability index. This indicates that the evaluated exercise program is more effective for subjects with LBP only for the outcome work ability.

### Impact of chronic low back pain on back pain related disability, trunk muscle strength, and work ability

Muscular strength of the back is a known parameter associated with non-specific LBP. Accordingly we observed that subjects with chronic LBP demonstrated less muscle strength for back extension at baseline. In addition, they had higher LBP-related disability as well as a less work ability compared to subjects without chronic LBP. These data confirm previous reports and underline the widespread impact of chronic LBP on global health and productivity [29]. Regular exercise is an established intervention for the management of LBP. In addition many other methods exist for preventing (e.g. education and stress reduction) or treating LBP (e.g. drugs, physical therapy, spinal manipulation, and surgery) [4, 5, 30, 31]. Without reservation insufficient or no evidence exist to recommend these interventions or prefer them to exercise therapy [32–35]. There is some evidence that programs targeting low back pain are more effective when they are individually supervised and high-dose in exercise volume [6]. However these programs require substantial effort and costs for those providing the work, questioning the transferability of results and usefulness of such programs for a majority of individuals.

### Effects of low-dose, non-supervised exercise on back strength and self-perceived pain

With a 5-month low-dose (60 min per week) and non-supervised exercise program, the employees’ muscle strengths for back extension and lateral flexion to the left increased. After stratification based on chronic LBP, exercise training improved these parameters only in the group of subjects with chronic LBP. The improvement in self-perceived pain was also limited to subjects who were already affected by chronic LBP (with 1.4 cm close to 1.5 cm stated as clinically important) [20]. Previous studies on changes in muscle strength and pain with low-dose interventions for patients with low back pain showed mixed results [7–12] demonstrating the need for further studies. The missing effect in subjects without chronic LBP might be a result of smaller increases in muscle strength and because their magnitude of self-estimated pain was already at a low level before the intervention. However, the exercise-induced improvements in muscle strength and low back pain were not significantly greater for subjects with LBP compared to those without LBP, indicating that the investigated exercise program is not more effective for subjects with chronic LBP for these outcomes. For the whole (non-stratified) study sample, we observed improvements in some outcomes within the control group (e.g. back extension strength or work ability). Possible reasons for that may include a learning effect for the assessment procedures (e.g. strength testing) and a self-reliant change in activity or nutrition habits due to inclusion in an exercise study.

### Chronic low back pain, exercise and ability to work

Lumbar pain is one of the most frequent and disabling condition affecting workers in their productive years [36]. Whereas physical exercise is considered as beneficial intervention for overall health and productivity in the general population, the effectiveness of exercise as a strategy to increase the work ability for employees with chronic back pain is less certain [37]. For the work ability index, which is a parameter associated with sickness absence and early retirement [38, 39], we observed no effect of our low-dose exercise program in the whole study sample. Previous studies on changes in work ability related factors after exercise reported inconsistent results [13, 40–43]. Our results are in line with some investigations suggesting that a single intervention is not sufficient and a multiprofessional approach is needed to improve work ability in the general workforce [40, 42, 44]. Nevertheless, we observed that work ability improved significantly after exercise training in subjects with LBP indicating that the evaluated exercise program might be particularly suitable for employees suffering from chronic LBP. Given the scarcity of data this issue warrants further studies. Of note, individuals with chronic LBP also experience more common cardiovascular, musculoskeletal and cerebrovascular disorders and diseases than do individuals without back pain [45]. Therefore, regular exercise for the prevention or treatment of LBP might be a useful component within an overall physically active lifestyle to also reduce sickness absence or early retirement by addressing common coexisting disorders in a beneficial manner [46–48].

### Personnel and financial demand of the health insurance conducted exercise program

A crucial question regarding the implementation of health interventions to a majority of persons is anticipated cost-effectiveness. The practical guidance of the evaluated program was conducted by two physiotherapists who were employed at the cooperating health insurance company with 50 and 75% of their working time. Based on the standard employment costs for this occupational group (3343 € per month) the investment per participant for the entire 20 weeks program was 190 €. Given an approximate value of 400 EUR for a single day of work, a supposed reduction of the intervention group’s work absence by 52 days (0.47 days per participant) would theoretically return the investment to the professionals who supervised the intervention. We observed different success in recruiting employees for the current intervention across the participating companies. Based on our current study experience a key factor of success was broad communication of the program in the company from the very beginning of its implementation. Another factor seems to be the already successfully completed health actions with the collaborating health insurance, which was evident in the company that was able to recruit the highest number of volunteers.

Our study has strengths and limitations. Strengths include having a relatively high number of participants, blinding of investigators assessing the primary endpoint, and providing the intervention for those who were initially allocated to the control group after the study, which likely contributed to the small number of drop-outs. Limitations are the lack of measures of adherence to the instructed exercises, an absence of detailed information about the workplaces, and the non-availability of actual sick days due to the data privacy policies of the companies.

In conclusion, in middle-aged employees a once per month-supervised low-dose exercise program for the trunk muscles resulted in a significant increase in back muscle strength, a significant decrease in low back pain and, for those with preexisting chronic low back pain, in improved work ability.

## Supporting information

S1 Dataset List of exercises. (PDF)

S2 Dataset Study raw data. (XLSX)

S1 Table Full estimation results of ANCOVA models. (DOCX)

S2 Table Detailed changes over time within subgroups stratified for preexisting LBP. (DOCX)

S1 Appendix CONSORT checklist. (DOC)

S2 Appendix Study protocol English language. (PDF)

S3 Appendix Study protocol German language. (PDF)
