# Supplementary material for: Low-dose, non-supervised, health insurance initiated exercise for the treatment and prevention of chronic low back pain in employees. Results from a randomized controlled trial
Source: PLoS One. 2017 Jun 29;12(6):e0178585. doi: 10.1371/journal.pone.0178585 (PMC5490969; doi:10.1371/journal.pone.0178585)
Supplement: S1 Dataset — (PDF) [file pone.0178585.s001.pdf]

Exercises

1

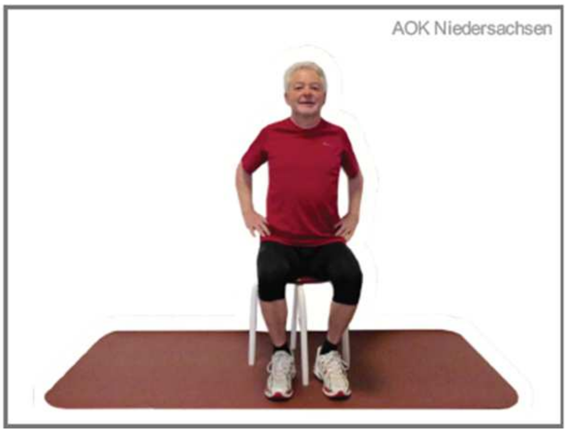

- Sit up straight on a chair
- Round your back and sit up straight again
- Press your shoulders down and move your elbow backwards (shoulder blades together)
- Hold this position for 15-20 seconds

Practicing: 3 rep., with 30 s pause between

2

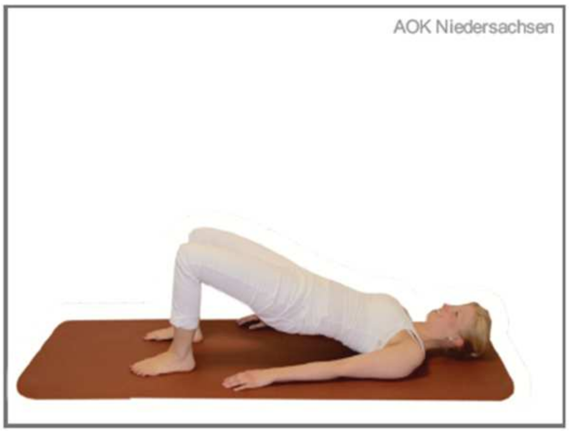

- Lie on your back with your arms by your sides and your head relaxed on the ground
- Bend your knees with your feet flat on the floor
- Now raise your hips to create a straight line from your knees to your chest, hold for 2-3 seconds
- Then slowly lower your back to the ground and repeat the exercise

Practicing: 10-12 rep., 2 sets

3

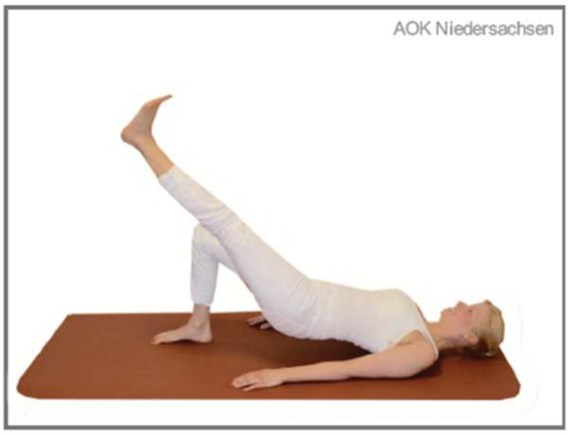

- Advanced exercise: same starting position with your feet flat on the ground and your knees bent at a right angle
- Lift up your hips to create a straight line from your knees to your chest
- From this position extend one leg briefly and then place its foot back next to the other feet
- Now extend the other leg and place it back
- Lower your hips to the ground and repeat the exercise
- Make sure your breathing slowly and regularly

Practicing: 8-10 rep., 2 sets

Exercises

4

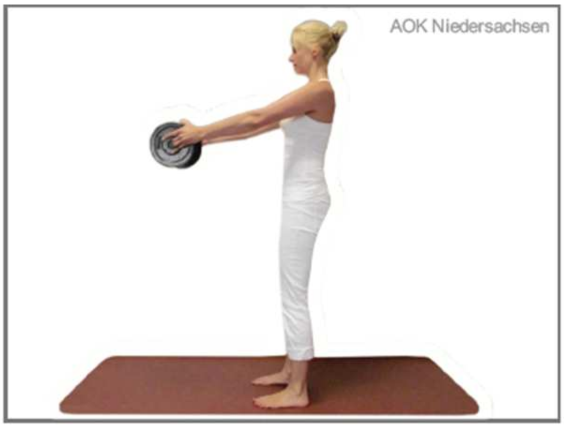

- Stand with your feet hip-width apart
- Keep your back straight (avoid a hollow-back)
- Hold an individual weight in each hand (e.g. a water bottle)
- Slowly move your arms up and down in front of you
- Alternative exercise: move your arms slowly up and down in opposite directions

Practicing: 10-12 rep., 3 sets

5

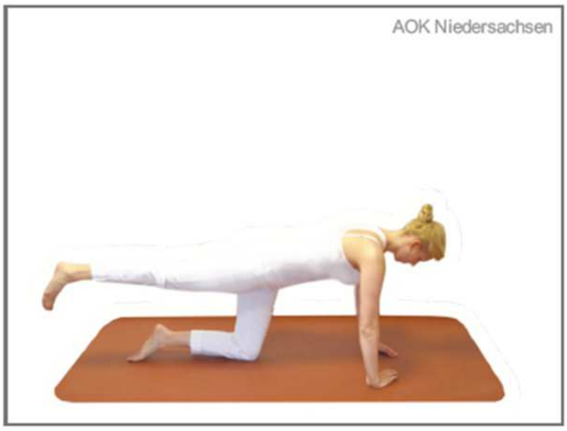

- Come to a hands and knees position (quadruped position)
- Head to a horizontal position, face the floor
- Extend one leg and bring it in line with your back (hold this position for 2 seconds)
- Keep your back straight (avoid a hollow back) by tensing your abdominal muscles
- Come back to the starting position and extend the other leg (hold for 2 seconds)

Practicing: 8-10 rep with both legs, 3 sets

6

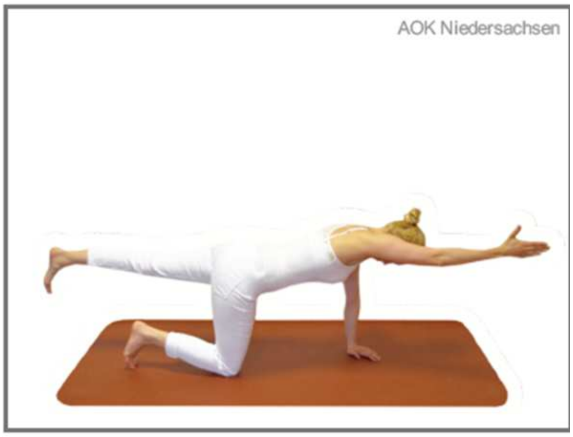

- From the same starting position (quadruped position): lift one arm at a horizontal position
- Hold this position for 2 seconds and make sure to keep your back straight and face the floor

Advanced exercise

- Extend one leg while lifting your diagonal arm and briefly hold this position
- For repetition change your arm and leg

Practicing: 8-10 rep with both sides, 3 sets

Exercises

7

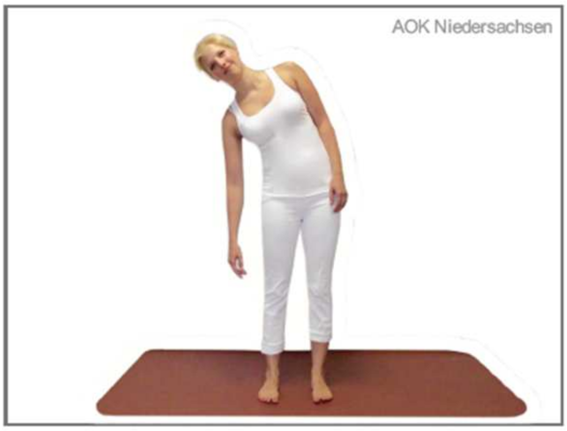

- Stand up comfortably
- Gently, tilt your body to the left and to the right side
- Hold it for a few seconds on each side
- Avoid evasive movements forwards or backwards

Practicing: 10 rep. on each side, 3 sets

8

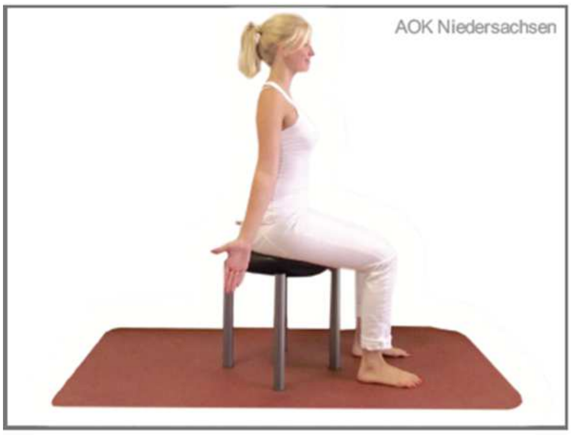

- Sit on a chair with your back straight and your feet flat on the floor
- Pull your shoulder blades together, with your arms going behind your torso (turn your palms outwards)
- Hold this position for 5 seconds

Practicing: 12 rep., 3 sets

9

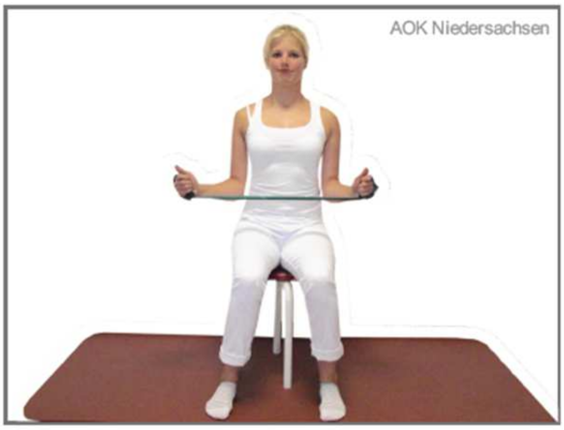

- Take an elastic band (or towel) and wrap it around your hands (no tension)
- Hold your arms at a right angle and keep your elbows close to your body
- Pull your hands apart at the same time(high tension), keep your elbows at your body
- Your hands come back to the middle, briefly relax and start again
- Make sure not to raise your shoulders

Practicing: slowly 10-12 rep., 3 sets

Exercises

10

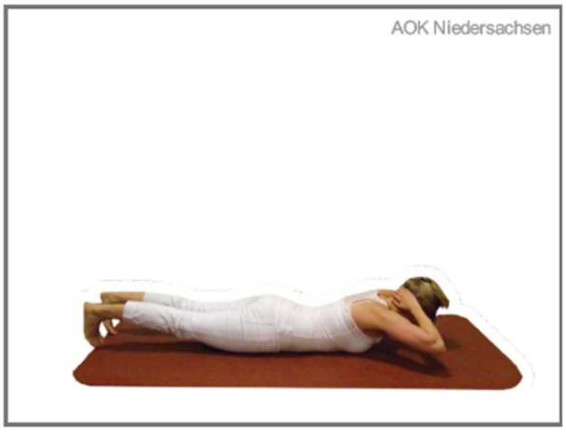

- Lie on your front with your legs stretched out
- Bend your elbows and lay your hands on your neck
- Hold this position briefly (for about 5 to 8 seconds)
- Lower your elbows and arms back to the ground, breathe one time and repeat

Practicing: 6-8 rep., 2 sets

11

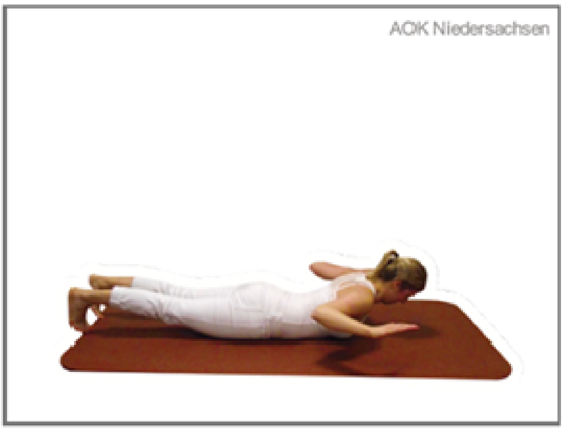

- Alternative exercise: same starting position
- Bend your elbows to approximately 90 degree
- Lift your arms and bring your shoulder blades together
- Hold this position briefly (for about 5 to 8 seconds)
- Advanced position: keep both arms straight over your head at a horizontal position

Practicing: 6-8 rep. for each exercise, 2 sets

12

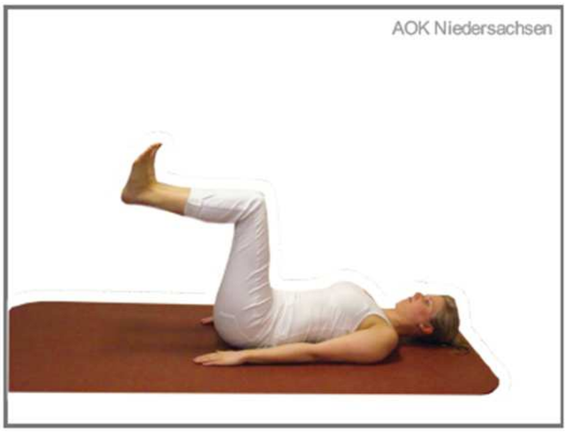

- Lie on your back at a bent knee position with your arms by your sides and your head relaxed on the floor
- Tense your abdominal muscles, optionally put your hands under your hips for stabilization
- Extend one leg and bring it in a cycling movement (3 to 5 times)
- Then perform with your other leg
- After that you can do it with both legs alternating at same time (cycling movement)

Practicing: "cycling" for 10-15 seconds, 3 sets

Exercises

13

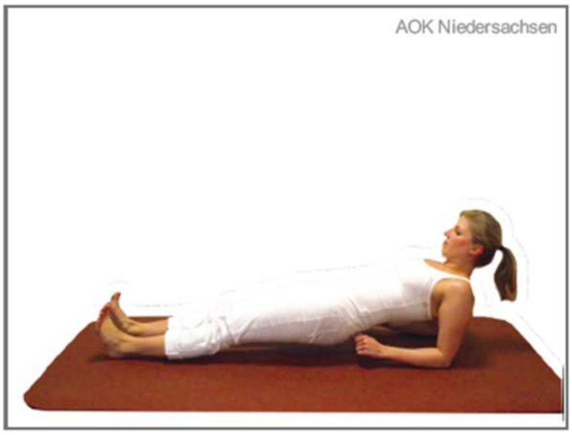

- Lie on your back and then lean on your elbows and forearms
- Raise your hips from the ground and hold this position for a few seconds
- Lower your hips to the ground and then repeat the exercise
- Make sure your breathing slowly and regularly

Practicing: 8-10 rep., 2 sets
