# Supplementary material for: Low-dose, non-supervised, health insurance initiated exercise for the treatment and prevention of chronic low back pain in employees. Results from a randomized controlled trial
Source: PLoS One. 2017 Jun 29;12(6):e0178585. doi: 10.1371/journal.pone.0178585 (PMC5490969; doi:10.1371/journal.pone.0178585)
Supplement: S1 Table — (DOCX) [file pone.0178585.s003.docx]

**Supporting Information**

**Full estimation results of our ANCOVA model for the primary and key secondary outcomes**

| **Parameter** | **Estimate** | **Standard Error** | **Pr > \|t\|** | **95% Confidence Limits** | |
| --- | --- | --- | --- | --- | --- |
| Intercept | 164.15 | 20.76 | <0.001 | 111.41 | 216.89 |
| Baseline strength back extension | -0.27 | 0.04 | <0.001 | -0.34 | -0.20 |
| **Company** |  |  |  |  |  |
| Company I | 71.15 | 20.91 | 0.046 | 12.81 | 129.48 |
| Company II | 29.09 | 23.59 | 0.219 | -17.40 | 75.58 |
| Company III | 0.00 | . | . | . | . |
| **Chronic low back pain** |  |  |  |  |  |
| Yes | -41.71 | 17.70 | 0.019 | -76.59 | -6.83 |
| No | 0.00 | . | . | . | . |
| **Group** |  |  |  |  |  |
| Exercise | 31.30 | 14.75 | 0.035 | 2.23 | 60.37 |
| Control | 0.00 | . | . | . | . |

**Table S1**: Results of the primary analysis with mean difference of isometric muscle strength for back extension (primary outcome) as response variable, and baseline strength for back extension, company, chronic low back pain and group as explanatory variables.

| **Parameter** | **Estimate** | **Standard Error** | **Pr >\|t\|** | **95% Confidence Limits** | |
| --- | --- | --- | --- | --- | --- |
| Intercept | 53.05 | 19.40 | 0.007 | 14.81 | 91.29 |
| Baseline strength lateral flexion to the left side | -0.17 | 0.04 | <0.001 | -0.25 | -0.09 |
| **Company** |  |  |  |  |  |
| Company I | 55.24 | 20.01 | 0.006 | 15.81 | 94.66 |
| Company II | 25.37 | 16.11 | 0.117 | -6.38 | 57.11 |
| Company III | 0.00 | . | . | . |  |
| **Chronic low back pain** |  |  |  |  |  |
| Yes | -12.39 | 11.98 | 0.302 | -35.99 | 11.21 |
| No | 0.00 | . | . | . | . |
| **Group** |  |  |  |  |  |
| Exercise | 27.06 | 10.13 | 0.008 | 7.09 | 47.03 |
| Control | 0.00 | . | . | . | . |

**Table S2**: Results of the primary analysis with mean difference of isometric muscle strength for lateral flexion to the left side (secondary outcome) as response variable, and baseline strength for lateral flexion to the left side, company, chronic low back pain and group as explanatory variables.

| **Parameter** | **Estimate** | **Standard Error** | **Pr > \|t\|** | **95% Confidence Limits** | |
| --- | --- | --- | --- | --- | --- |
| Intercept | 50.22 | 22.23 | 0.025 | 6.41 | 94.02 |
| Baseline strength lateral flexion to the right side | -0.10 | 0.04 | 0.030 | -0.18 | -0.01 |
| **Company** |  |  |  |  |  |
| Company I | 41.63 | 23.26 | 0.075 | -4.22 | 87.48 |
| Company II | 42.01 | 18.61 | 0.025 | 5.33 | 78.68 |
| Company III | 0.000 | . | . | . | . |
| **Chronic low back pain** |  |  |  |  |  |
| Yes | -25.90 | 13.69 | 0.060 | -52.87 | 1.07 |
| No | 0.00 | . | . | . | . |
| **Group** |  |  |  |  |  |
| Exercise | 18.73 | 11.69 | 0.111 | -4.32 | 41.77 |
| Control | 0.00 | . | . | . | . |

**Table S3**: Results of the primary analysis with mean difference of isometric muscle strength for lateral flexion to the right side (secondary outcome) as response variable, and baseline strength for lateral flexion to the right side, company, chronic low back pain and group as explanatory variables.

| **Parameter** | **Estimate** | **Standard Error** | **Pr > \|t\|** | **95% Confidence Limits** | |
| --- | --- | --- | --- | --- | --- |
| Intercept | 0.70 | 0.37 | 0.058 | -0.03 | 1.43 |
| Baseline low back pain | -0.50 | 0.05 | <0.001 | -0.60 | -0.41 |
| **Company** |  |  |  |  |  |
| Company I | 0.09 | 0.44 | 0.84 | -0.78 | 0.96 |
| Company II | 0.20 | 0.36 | 0.58 | -0.51 | 0.92 |
| Company III | 0.00 | . | . | . | . |
| **Chronic low back pain** |  |  |  |  |  |
| Yes | 1.41 | 0.30 | <0.001 | 0.82 | 1.99 |
| No | 0.00 | . | . | . | . |
| **Group** |  |  |  |  |  |
| Exercise | -0.72 | 0.23 | 0.002 | -1.17 | -0.27 |
| Control | 0.00 | . | . | . | . |

**Table S4:** Results of the primary analysis with mean difference of low back pain on a visual analogue scale (secondary outcome) as response variable, and baseline low back pain, company, chronic low back pain and group as explanatory variables.

| **Parameter** | **Estimate** | **Standard Error** | **Pr > \|t\|** | **95% Confidence Limits** | |
| --- | --- | --- | --- | --- | --- |
| Intercept | 12.21 | 1.80 | <0.001 | 8.66 | 15.76 |
| Baseline low back pain | -0.28 | 0.04 | <0.001 | -0.36 | -0.20 |
| **Company** |  |  |  |  |  |
| Company I | 0.24 | 0.80 | 0.764 | -1.33 | 1.82 |
| Company II | -0.72 | 0.66 | 0.270 | -2.01 | 0.57 |
| Company III | 0.00 | . | . | . | . |
| **Chronic low back pain** |  |  |  |  |  |
| Yes | -0.42 | 0.52 | 0.418 | -1.44 | 0.60 |
| No | 0.00 | . | . | . | . |
| **Group** |  |  |  |  |  |
| Exercise | 0.53 | 0.41 | 0.199 | -0.28 | 1.35 |
| Control | 0.00 | . | . | . | . |

**Table S5**: Results of the primary analysis with mean difference of work ability (secondary outcome) as response variable, and baseline work ability, company, chronic low back pain and group as explanatory variables.
