# Supplementary material for: Low-dose, non-supervised, health insurance initiated exercise for the treatment and prevention of chronic low back pain in employees. Results from a randomized controlled trial
Source: PLoS One. 2017 Jun 29;12(6):e0178585. doi: 10.1371/journal.pone.0178585 (PMC5490969; doi:10.1371/journal.pone.0178585)
Supplement: S2 Table — (DOCX) [file pone.0178585.s004.docx]

**Supporting Information**

**Detailed changes over time within subgroups stratified for preexisting LBP**
