# Supplementary material for: Low-dose, non-supervised, health insurance initiated exercise for the treatment and prevention of chronic low back pain in employees. Results from a randomized controlled trial
Source: PLoS One. 2017 Jun 29;12(6):e0178585. doi: 10.1371/journal.pone.0178585 (PMC5490969; doi:10.1371/journal.pone.0178585)
Supplement: S2 Appendix — (PDF) [file pone.0178585.s006.pdf]

Study protocol

Title

Evaluation of effects of an exercise program on back-health

Principal investigator
Dr. Sven Haufe
Institute for Sports Medicine/ Institute for Clinical Pharmacology
Hannover Medical School
Carl-Neuberg-Straße 1
30625 Hannover

CLINICAL TRIAL OUTLINE APPLICATION

1. STUDY SYNOPSIS

**APPLICANT / COORDINATING INVESTIGATOR**	Dr. Sven Haufe<br>Institute of Sports Medicine and Institute of Clinical Pharmacology<br>Tel.: 0511 5325499, Fax: 0511 5328199
**TITLE OF STUDY**	Evaluation of effects of an exercise program on back-health
**CONDITION**	Healthy participants, as well as persons with chronic low back pain.
**OBJECTIVE(S)**	Evaluation of effects of a health-insurance initiated program for back-health in companies.
**INTERVENTIONS(S)**	<u>Intervention</u>:<br>Structured training to strengthen (vitalize) back muscles (3 days per week for 20 min.) over a period of 20 weeks.<br><br><u>Control group</u>:<br>20 weeks no intervention as waiting-control group, followed by a training intervention over a period of 20 weeks (see intervention)<br><br><u>Duration of intervention per patient</u>:<br>20 weeks<br><br><u>Follow-up per patient</u><br>Not intended
**KEY INCLUSION AND EXCLUSION CRITERIA**	<u>Inclusion criteria</u>:<br>- Male and female<br>- Age: ≥ 18 to 67 years<br><br><u>Exclusion criteria</u>:<br>- Actual participation of training for back-health<br>- Known alcohol or drug abuse<br>- Diseases which exclude a participation<br>- Clinical relevant, acute or chronic infections<br>- Pregnant women
**OUTCOME(S)**	<u>Primary endpoint</u>:<br>- Isometric low back strength (newton meter in 0 degree extension)<br><br><u>Key secondary endpoints</u>:<br>- Isometric strength of lateral flexion<br>- Change of back pain score with the Oswestry Low Back Pain Disability Questionnaire (OBQ)<br>- Visual analog scale (VAS)<br>- Evaluation of the health-related quality of life by the questionnaire SF-36<br>- Work-Ability-Index<br>- “Freiburger” activity questionnaire<br>- Sick days<br><br><u>Documentation for safety</u>:

	Documentation of any serious events
**STUDY TYPE**	Prospective, randomized and controlled study.
**STATISTICAL ANALYSIS**	Effectivity:<br>The primary goal of the study is to show that a structured training to strengthen the back-muscles over 20 weeks results in an improvement of isometric low back strength in comparison to the control group. The two-sided type1 error is defined as 5%.<br><br>Description of the statistic evaluation of the primary endpoint<br>The primary evaluation is carried out in the intention-to-treat (ITT) population. For back strength in newton meter an ANCOVA-Model will be used with the before-after difference as dependent-variable. Important co-variables are baseline strength, the group (intervention or control), low-back pain (yes/no) as well as the company. The intervention is successful, if the lower mark of the two-sided 95%-confidence interval is bigger than 0.<br><br>Secondary endpoints<br>The most important secondary endpoints will be evaluated as the primary endpoint. For dichotomic endpoints a logistic regression will be used and adjusted for the same co-variables. 95%-confidence interval will be calculated for all secondary endpoints.<br><br>Safety and incidences<br>Any unexpected event of randomized participants will be captured and descriptively compared between the groups.
**SAMPLE SIZE**	Tested for eligibility: **n = 250 per group**<br>Randomized for participation: **n = 200 per group**<br>To analyze : **n = 200 per group**
**TRIAL DURATION**	Duration first participant in and last participant out(weeks): 50<br>Duration of whole study (weeks): 52<br>Recruiting period (weeks): 10
**PARTICIPATING CENTRES**	Participating companies = 3<br>Study administration: Institute of Sports Medicine, MHH

2. THE MEDICAL PROBLEM

2.1 EVIDENCE

Low-back pain is still the most common kind of pain in the population. Approximately one-third of men and women suffer from chronic dorsalgia (see fig. 1).
Back pain can be divided in specific and non-specific pain. Specific back pain has an explicit diagnosable cause, for example herniated disks, spinal canal stenosis, vertebral body fractions, tumors, infections or inflammatory diseases. Non-specific dorsalgia has no physiological diagnosable cause. 15 percent of all patients with dorsalgia have specific pain, 85 percent have nonspecific pain, which heavily complicates the treatment.

	Frauen		Männer	
	2003	2009	2003	2009
18–29 Jahre	20,8%	20,8%	12,8%	12,8%
30–39 Jahre	26,1%	27,8%	19,9%	20,5%
40–49 Jahre	29,0%	31,5%	23,3%	26,1%
50–59 Jahre	33,8%	39,1%	33,4%	33,8%
60–69 Jahre	34,5%	41,8%	32,8%	35,1%
70 Jahre u. älter	36,4%	46,7%	30,0%	36,1%

Fig. 1: Dorsalgia (minimum 3 month, almost daily) of the German population
Source: Robert Koch Institut, Abb. Zum Themenheft 53 - Rückenschmerzen

Consequences of dorsalgia besides restricted subjective health and reduced productivity are also industrial accidents plus early pension. Backache is still the cause for every tenth day of incapacity to work in Germany.
In the ranking of top 10 diseases with the longest period of disability dorsalgia (ICD-10-GM: M54) is on the first place amongst AOK-members (without pensioners) with 14.5 million days of incapacity to work.
This equates the percentage of 7.0 %. Per case there are 11.7 days of incapacity to work (women 12.2 days, men 11.4 days). Barmer GEK (health insurance) also has dorsalgia on the first position of their ranking of days of incapacity to work from 2009 (round about 6.5 % of all days of incapacity to work) (2)

2.2 THE NEED FOR A TRIAL

Accordingly high is the claiming of ambulant and stationary medical care because of back pain (see Fig. 2). The absence from work as well as the costs for the health insurances display an actual and future significant socio-economic problem in Germany.

	Bevölkerungsprävalenz			Anteile derjenigen mit Rückenschmerzen in den vorangegangenen 12 Monaten		
	Frauen	Männer	Gesamt	Frauen	Männer	Gesamt
Rückenschmerzen in den letzten 12 Monaten	65,8 %	57,4 %	61,8 %			
Arztbesuch wegen Rückenschmerzen*	28,6 %	22,5 %	25,7 %	43,5 %	39,1 %	41,5 %
Ambulant*	27,8 %	21,7 %	24,8 %	42,2 %	37,7 %	40,2 %
Stationär*	2,4 %	3,1 %	2,8 %	3,7 %	5,4 %	4,5 %
Reha (inkl. AHB) wegen Rückenschmerzen**	4,8 %	5,4 %	5,1 %	7,2 %	9,4 %	8,2 %

* in den letzten 12 Monaten; **jemals
AHB: Anschlussheilbehandlung

Fig. 2 Prevalence of back pain and medical treatment because of dorsalgia
Source: Kohler M, Ziese T (2004) Telefonischer Gesundheitssurvey. Ein Wort des Robert Koch-Instituts zu chronischen Krankheiten und ihren Bedingungen. RKI, Berlin

Dorsalgia is still a widespread disease that mostly accrues because of one-sided exposure and sedentary. Sports and exercises have to be inherent parts for the therapy of dorsalgia, ideally also for prevention.

Promising is the concept of the so called segmental stabilization. Segmental stabilization means to strengthen the individual movement segments of the spine. Primarily responsible for that are the deep-seated muscles of the spine (e.g. muscle M. transversus abdominis and the M. multifidus lumbalis). Those are no big or strong muscles, but sensible muscles that put single parts of the vertebral body in the biomechanical right position to protect intervertebral disc in case of wrong exposure. In the review of Rackwitz (2006) (3) the segmental stabilization exercises (SSE), were evaluated as reference to acute, subacute and chronical dorsalgia (low back pain). The parameters are pain, the disability caused by dorsalgia and the return to the workplace. The review has following outcome: by acute dorsalgia, SSE is for short-term pain lowering and incapacity as effective as medical treatment (general practitioner). To achieve long-term pain lowering segmental stabilization exercises are more effective than medical treatment. In case of chronical dorsalgia SSE are on short- and long-term more effective than medical treatment (general practitioner) to reduce pain and incapacity.

Certainly this training isn't studied enough in terms of its effectiveness on the strength of the back muscles and the ability to work of employees in companies. Therefor the effects of instructed but independent executed 20-week training with focus on SSE, in a larger group with and without unspecific lumbar dorsalgia will be tested in this study.

References

1. Wissenschaftliches Institut der AOK (WIdO) (2011) Die 10/20/50 Erkrankungen mit den längsten Arbeitsunfähigkeitszeiten in Tagen bei AOK-Pflichtmitgliedern ohne Rentner. WIdO, Berlin)

2. BARMER GEK (Hrsg) (2010) Gesundheitsreport 2010. Teil 1. Gesundheitskompetenz in Unternehmen stärken, Gesundheitskultur förder, www.barmer-gek.de/barmer/web/Portale/Presseportal/Subportal/Infothek/Studien und Reports/ Gesundheitsreport 2010/Teil-1-AU-Daten/Gesundheitsreport- 2010-PDF, property=Data.pdf (Stand: 24.10.2012)

3. Rackwitz B, Bie RD, Limm H, Garnier KV, Ewert T, Stucki G (2006) Segmental stabilizing exercises and low back pain. What is the evidence? A systematic review of randomized controlled trials. Clinical Rehabilitation. 20:553

3. JUSTIFICATION OF DESIGN ASPECTS

3.1 CONTROL(S) / COMPARATOR(S)

The intervention group will get an individual instruction for the training of the back muscles by physiotherapists after the screening examination. The exercises will be educated, controlled and adjusted by the trainer through regular meetings (once per month). The educated exercises should be executed independently on 3 days per week, each 20 min. Additionally employees of the AOK will search for fitting primary and secondary preventive activities for back-health and increasing of physical activity in everyday life, and offer those to participants.

The control group won't have any intervention (usual care) and will be asked to keep their actual lifestyle. After the control period, those participants will do the 20 week intervention (waiting control group).

3.2 INCLUSION / EXCLUSION CRITERIA

Inclusion criteria:

- Men and women
- Age: ≥ 18 to 67 years

Exclusion criteria:

- Actual participation of training for back-health
- Known alcohol or drug abuse
- Diseases which exclude a participation
- Clinical relevant, acute or chronic infections
- Pregnant women

3.3 OUTCOME MEASURES

Key primary endpoint:

- Isometric low back strength (newton meter in 0 degree extension) as the post-pre difference

Key secondary endpoints:

- Isometric strength of lateral flexion to the left and right
- modification of dorsalgia with help of the Oswestry low back pain disability questionnaire (OBQ)
- Visual analog scale (VAS)
- Determination of the health claiming life quality by using the questionnaire SF-36
- Work-Ability-Index
- Freiburger activity questionnaire
- sick days

3.4 METHODS AGAINST BIAS

This is a prospective, randomized-controlled, and observer- blinded study. An exercise instructor will take control over the tests of the back strength before and after the 20 week intervention. He does not get any information about the group allocation of subjects by randomization. The training requirements with instruction will be executed by a different exercise instructor. It will be a central randomization stratified by the company as well as the health status of the subscriber (back pain and no back pain), for reason that these variables have a big impact of the primary end point. The evaluation of the primary and the most important secondary end points is made

after the intention-to-treat-principle (ITT principle) to avoid an overestimation of the effect through drop-outs. Missing values will be conservatively replaced. The baseline-observation-carried-forward method (BOCF) is used for back strength measured in Newton meter.

3.5 PROPOSED SAMPLE SIZE / POWER CALCULATIONS

The primary goal of the study is to show that an improvement of the isometric low back strength can be achieved after 20 weeks of training. The back strength is measured in newton meter, in which case high values are positive. By looking at the after-before differences, positive trunk strength-differences indicate an improvement of the functional back health. We plan to include 200 volunteers. Therefore the planning is calculated in nQuery Advisor 7.0, which effect could be shown by given number of subjects. The number of evaluable patients is based on data of an prospective, randomized study with 21 volunteers from Moon HJ et al., Ann Rehabil Med., 2013, 37(1): 110-117, in which two intervention methods are being executed on patients with chronical back pain for a period of eight weeks. In the group with a similar training comparable to the planed intervention a mean improvement from 104.0 (sd=42.5) to 135.2 (sd=75.9) could be observed. Assuming that the control group remains similar and both groups start with the same baseline-values (average= 104.0), it comes to the result that with a t-test for same variances (bigger variance to conservative planning (sd= 75.9)), a 1. Type error of 5 % (double sided) and a power of 80%, an improvement up to 134.22 Newton meter can be expected. This correlates roughly to the change which Moon et al. has been shown and is considered relevantly so that the number of cases can be considered as adequate.

3.6 FEASIBILITY OF RECRUITMENT

The recruiting of volunteers for the AOK prevention program for back health will take place in three companies over an internal informative events and internal advertisement. The number of employees in these companies is all in all about 2000 employees. Due to the expectedly positive effects of the event and the degree of esteem of training to strengthen the musculoskeletal system, we are not expecting any problems referring to the recruitment. We expect that the planned 200 volunteers will be recruited.

4. STATISTICAL ANALYSIS

<u>Design</u>

The planned study is a randomized-controlled, and observer-blinded study. Volunteers from three different companies, which conform to the inclusion and exclusion criteria, will be randomized into an intervention- or waitlist control group central 1:1 after screening. The randomization will be stratified after back pain (yes versus no) as well as the company, which is an important influence. Subsequent to that the waitlist- control group receives a 20 week long Intervention phase the same training. In both groups will be raised the primary final point of the trunk strength in newton meter and the secondary final point before and after the intervention.

<u>Primary analyses:</u>

For the analysis of the primary end point of low-back strength, measured in newton, a ANCOVA-model, with a mean after-before difference, is used as the variable of interest. Important influences are the baseline-values of low-back strength, the groups (intervention or waitlist-control group), back pain (yes/no) and the company. The intervention is successful, when the lower boundary of the two-sided 95%- confidence interval is bigger than 0.

<u>Auswertung der sekundären Endpunkte</u>

The most important secondary end points will be analyzed as same as the primary end points. For dichotomous end points a logistic regression, adjusted for the same co- variables, will be used. For all secondary endpoints 95% confidence intervals will be calculated.

Safety Analysis

Unexpected events will be documented as absolute und relative numbers. The comparison of groups will be analyzed with the Chi-quadrat test and p-values will be considered descriptively.

5. ETHICAL CONSIDERATIONS

There are no specific risks by the conduction of this study.

6 TRIAL MANAGEMENT

6.1 MAJOR PARTICIPANTS

	Name	Affiliation	Responsibility / Role	Signature
1	Dr. Sven Haufe	Institution of sports medicine Institution of clinical pharmacology	Principal investigator	
2	Prof. Dr. Uwe Tegtbur	Institution of sports medicine	Co-investigator	
3	Dr. Arno Kerling	Institution of sports medicine	Co-investigator	
4	Prof. Dr. Armin Koch	Institution of biometry	Statistician	
5	Andrea Gonnermann	Institution of biometry	Statistician	
6	Lothar Stein	Institution of sports medicine	Co-investigator	
7	Momme Kück	Institution of sports medicine	Co-investigator	

6.2 TRIALS EXPERTISE

Das Studienteam vereint alle für das Projekt notwendigen Kompetenzen.
Dr. Sven Haufe, Sportwissenschaftler, Studienleiter und Autor zahlreicher Publikationen, Erfahrung in der Durchführung klinischer Studien und in der Betreuung von Mitarbeitern in der betrieblichen Prävention.
Prof. Dr. Uwe Tegtbur, Direktor des Instituts für Sportmedizin, Sportmediziner, hat Erfahrung in der Leitung und Durchführung von Studien zur betrieblichen Mitarbeiterprävention (u.a. Fit for Work Life als disziplinübergreifendes MHH-Präventionsangebot für Mitarbeiter, auch Kurse entsprechend dem Leitfaden Prävention sowie Rebirth-active II). Er ist Leiter des vom Deutschen Olympischen Sportbund lizenzierten Untersuchungszentrums in Niedersachsen, und erfüllt damit der Qualitätsrichtlinien für die sportmedizinische Diagnostik.
Dr. Arno Kerling, Oberarzt des Instituts für Sportmedizin, Sportmediziner. Vorerfahrung s. o.g. Studien, verantwortlicher Oberarzt für >1000 komplexe leistungsdiagnostische, sportmedizinische Untersuchungen
Lothar Stein, Sportwissenschaftler, M.A., Diplomtrainer, Vorerfahrung in der Planung und Durchführung der o.g. Studien und Projekte
H. Momme Kück; Expertise in der Studienplanung, -dokumentation und –analyse, seit 8 Jahren für o.g. wissenschaftliche Projekte.

The study team involves every important competence, which is relevant for the project. Dr. Sven Haufe, sports scientist, principal investigator and author of many publications, has experiences in the execution of clinical studies and the support of employees in the workplace prevention.
Prof. Dr. Uwe Tegtbur, director of the Institute of sports medicine, has experiences in the direction and execution of studies referring to internal employee prevention (fit for work and life as an interdisciplinary MHH-prevention offer for employees, also as courses appropriating the guideline prevention as well as rebirth- active II). He is the leader of the “Deutschen Olympischen Sportbund” licensed Olympic sport center center in lower Saxony und fulfills therefore quality guidelines referring to sports medical diagnostics.

Dr. Arno Kerling, assistant medical director of the institute of sports medicine, has also knowledge in the mentioned above studies and is responsible assistant medical director for > 1000 complex diagnostically performances per year, and sports medical investigation
Lothar Stein, sports scientist, MA, diploma trainer, previous knowledge in planning and execution of the above named studies and projects.
H. Momme Kück, Expertise in study planning, - documentation and analysis since 8 years for the above named academic projects.

Selected publications

Kerling A, Tegtbur U, Ziegenbein M, Grams L, Heinze DR, Sieberer M. Exercise Capacity and Quality of Life in Patients with Schizophrenia. Psychiatr Q. 2013 Mar 3. [Epub ahead of print] PubMed PMID: 23456450.

Helmer A, Kretschmer F, Deparade R, Song B, Meis M, Hein A, Marschollek M, Tegtbur U. A system for the model based emergency detection and communication for the telerehabilitation training of cardiopulmonary patients. Conf Proc IEEE Eng Med Biol Soc. ;2012:702-6. doi: 10.1109/EMBC.2012.6346028. PubMed PMID:23365989.

Dierich M, Tecklenburg A, Fuehner T, Tegtbur U, Welte T, Haverich A, Warnecke G, Gottlieb J. The influence of clinical course after lung transplantation on rehabilitation success. Transpl Int. 2013 Mar;26(3):322-30. doi: 10.1111/tri.12048. Epub 2013 Jan 7. PubMed PMID: 23294442.

Tegtbur U, Busse MW, Kubis HP. [Exercise and cellular adaptation of muscle]. Unfallchirurg. 2009 Apr;112(4):365-72. doi: 10.1007/s00113-009-1627-9. Review. German. PubMed PMID: 19308345.

Plischke M, Marschollek M, Wolf KH, Haux R, Tegtbur U. CyberMarathon - increasing physical activity using health-enabling technologies. Stud Health Technol Inform. 2008;136:449-54. PubMed PMID: 18487772.

6.3 TRIAL-SUPPORTING FACILITIES

- not applicable

7 FINANCIAL SUMMARY

**Item**	**Total funding period (€)**
Clinical project management	
Project management	
Case payment	
Data management	
Biostatistics	
Quality assurance	
Travel	
Materials	
Trial drug	
Fees, insurance	
Other	
**TOTAL**	

The study specific work is partially funded by AOK health insurance

Co-financing of the trial by a company:

See above
