# Supplementary material for: Low-dose, non-supervised, health insurance initiated exercise for the treatment and prevention of chronic low back pain in employees. Results from a randomized controlled trial
Source: PLoS One. 2017 Jun 29;12(6):e0178585. doi: 10.1371/journal.pone.0178585 (PMC5490969; doi:10.1371/journal.pone.0178585)
Supplement: S3 Appendix — (PDF) [file pone.0178585.s007.pdf]

Ethikantrag

Titel

Evaluation der Effekte einer Präventionsmaßnahme zur Rückengesundheit.

Studienleiter
Dr. Sven Haufe
Institut für Sportmedizin/ Institut für Klinische Pharmakologie
Medizinische Hochschule Hannover
Carl-Neuberg-Strasse 1
30625 Hannover

CLINICAL TRIAL OUTLINE APPLICATION

1. STUDY SYNOPSIS

**APPLICANT / COORDINATING INVESTIGATOR**	Dr. Sven Haufe<br>Institute für Sportmedizin und Institut für Klinische Pharmakologie<br>Tel.: 0511 5325499, Fax: 0511 5328199
**TITLE OF STUDY**	Evaluation der Effekte einer Präventionsmaßnahme zur Rückengesundheit.
**CONDITION**	Gesunde Probanden, sowie Personen mit chronisch-intermittierenden oder akuten Rückenbeschwerden
**OBJECTIVE(S)**	Evaluation der Effekte einer AOK-Präventionsmaßnahme zur Rückengesundheit in Betrieben.
**INTERVENTIONS(S)**	<u>Intervention</u>:<br>Strukturiertes Training zur Kräftigung der Rückenmuskulatur (3 Tage pro Woche a 20 min.) über 20 Wochen.<br><br><u>Kontrollgruppe</u>:<br>20 Wochen keine Intervention als Warte-Kontrollgruppe, gefolgt von einer Trainingsintervention über 20 Wochen (siehe Intervention)<br><br><u>Dauer der Intervention pro Patient</u>:<br>20 Wochen<br><br><u>Nachbeobachtung pro Patient</u><br>Nicht vorgesehen
**KEY INCLUSION AND EXCLUSION CRITERIA**	<u>Einschlusskriterien</u>:<br>- Männer und Frauen<br>- Alter: ≥ 18 bis 67 Jahre<br><br><u>Ausschlusskriterien</u>:<br>- aktuelle Teilnahme an einem Programm zur Rückengesundheit<br>- bekannter Alkohol- oder Drogenmissbrauch<br>- Krankheiten welche die Teilnahme an einer Trainingsintervention ausschließen<br>- klinisch relevante akute oder chronische Infektionen<br>- schwangere Frauen
**OUTCOME(S)**	<u>Primärer Endpunkt</u>:<br>- isometrische Messung der Kraft der Rumpfmuskulatur (Newtonmeter in 0 Grad Extension)<br><br><u>Key secondary endpoints</u>:<br>- isometrische Messung der Kraft bei Lateralflexion<br>- Änderung des Rückenschmerzscores über den Oswestry Low Back Pain Disability Questionnaire (OBQ)<br>- Visual analog scale (VAS)<br>- Ermittlung der gesundheitsbezogenen Lebensqualität durch den Fragebogen SF-36

	- Work-Ability-Index<br>- Freiburger Aktivitätsfragebogen<br>- Fettmasse und fettfreie Masse<br>- Fehl- und Krankheitstage<br><br><u>Dokumentation zur Sicherheit</u>:<br>Dokumentation jeglicher schwerwiegender Ereignisse
**STUDY TYPE**	Prospektive, randomisierte und kontrollierte Studie.
**STATISTICAL ANALYSIS**	<u>Wirksamkeit:</u><br>Das primäre Ziel der Studie ist es zu zeigen, dass durch ein strukturiertes Training zur Kräftigung der Rückenmuskulatur über 20 Wochen eine Verbesserung der isometrischen Rumpfkraft erreicht werden kann im Vergleich zur Wartelisten-Kontrollgruppe. Der zweiseitige Fehler 1. Art wird auf 5% festgelegt.<br><br><u>Beschreibung der statistischen Auswertung des primären Endpunkts</u><br>Die primäre Auswertung folgt dem intention-to-treat (ITT) Prinzip. Für die Rumpfkraft in Newtonmeter wird ein ANCOVA-Modell verwendet mit der mittleren Nachher-Vorher Differenz als Zielvariable. Wichtige Einflussvariablen sind die Baseline-Werte, die Gruppen (Intervention oder Wartelisten-Kontrolle), Rückenbeschwerden (ja/nein) sowie der Betrieb. Die Intervention hat Erfolg, wenn die untere Grenze des zweiseitigen 95%-Konfidenzintervalls größer als 0 ist.<br><br><u>Sekundäre Endpunkte</u><br>Die wichtigsten sekundären Endpunkte werden wie der primäre Endpunkt ausgewertet. Für dichotome Endpunkte wird die logistische Regression, adjustiert für dieselben Ko-variablen, verwendet. Für alle sekundären Endpunkte werden 95%-Konfidenzintervalle berechnet<br><br><u>Sicherheit und Ereignisse</u><br>Jegliche unerwünschten Ereignisse werden bei allen randomisierten Probanden erfasst und zwischen den Gruppen deskriptiv verglichen
**SAMPLE SIZE**	<u>Geprüft für Eignung zur Teilnahme</u>: **n = 250 pro Gruppe**<br><u>Randomisiert zur Teilnahme</u>: **n = 200 pro Gruppe**<br><u>Auszuwerten</u> : **n = 200 pro Gruppe**
**TRIAL DURATION**	<u>Einschluss erster zu Ausschluss letzter Proband (Wochen)</u>: 50<br><u>Dauer der gesamten Studie (Wochen)</u>: 52<br><u>Rekrutierungsdauer (Wochen)</u>: 10
**PARTICIPATING CENTRES**	Teilnehmende Betriebe = 3<br>Studienleitung: Institut für Sportmedizin, MHH

2. THE MEDICAL PROBLEM

2.1 EVIDENCE

Rückenschmerz ist nach wie vor die häufigste Schmerzform in der Bevölkerung. Etwa ein Drittel aller Frauen und Männer leiden unter chronischen Rückenschmerzen (siehe Abb. 1)
Rückenschmerzen lassen sich in spezifische und nichtspezifische Schmerzen unterteilen. Spezifische Rückenschmerzen haben eine eindeutig feststellbare Ursache, darunter fallen beispielsweise Bandscheibenvorfälle, Wirbelgleiten, Spinalkanalverengungen, Wirbelkörperbrüche, Tumore, Infektionen oder entzündliche Erkrankungen. Nichtspezifische Rückenschmerzen haben keine physiologisch erklärbare Ursache. 15 Prozent aller Rückenschmerzpatienten haben spezifische Schmerzen, bei 85 Prozent ist die Ursache ungeklärt, was die Behandlung erheblich erschwert.

	Frauen		Männer	
	2003	2009	2003	2009
18–29 Jahre	20,8%	20,8%	12,8%	12,8%
30–39 Jahre	26,1%	27,8%	19,9%	20,5%
40–49 Jahre	29,0%	31,5%	23,3%	26,1%
50–59 Jahre	33,8%	39,1%	33,4%	33,8%
60–69 Jahre	34,5%	41,8%	32,8%	35,1%
70 Jahre u. älter	36,4%	46,7%	30,0%	36,1%

Abb. 1: Rückenschmerzen (mind. 3 Monate, fast täglich) in der deutschen Bevölkerung
Quelle: Robert Koch Institut, Abb. Zum Themenheft 53 - Rückenschmerzen

Folgen der Rückenschmerzen sind neben der eingeschränkten subjektiven Gesundheit und der verminderten Leistungsfähigkeit auch Arbeitsausfälle sowie Frühberentungen. Rückenbeschwerden sind nach wie vor die Ursache für jeden zehnten Krankschreibungstag in Deutschland.
In der Rangliste der zehn Erkrankungen mit den längsten Arbeitsunfähigkeitszeiten liegen die Rückenschmerzen (ICD-10-GM: M54) im Jahr 2010 unter den AOK-Pflichtmitgliedern (ohne Rentner) mit 14,5 Millionen Arbeitsunfähigkeitstagen (AU-Tage) auf dem ersten Rang (1)
Dies entspricht einem Anteil von 7,0 %. Pro Fall ergeben sich 11,7 AU-Tage (Frauen 12,2 AU-Tage, Männer 11,4 AU-Tage) Auch bei der Barmer GEK liegen die Rückenschmerzen 2009 auf dem ersten Rang der Arbeitsunfähigkeits-Statistiken (mit rund 6,5 % aller AU-Tage) (2)

2.2 THE NEED FOR A TRIAL

Entsprechend ist die Inanspruchnahme ambulanter und stationärer ärztlicher Versorgung aufgrund von Rückenschmerzen hoch (siehe Abb. 2). Die Fehlzeiten am Arbeitsplatz sowie die Kosten der Krankenkassen stellen ein aktuelles und für die Zukunft bedeutendes sozioökonomisches Problem in Deutschland dar.

	Bevölkerungsprävalenz			Anteile derjenigen mit Rückenschmerzen in den vorangegangenen 12 Monaten		
	Frauen	Männer	Gesamt	Frauen	Männer	Gesamt
Rückenschmerzen in den letzten 12 Monaten	65,8 %	57,4 %	61,8 %			
Arztbesuch wegen Rückenschmerzen*	28,6 %	22,5 %	25,7 %	43,5 %	39,1 %	41,5 %
Ambulant*	27,8 %	21,7 %	24,8 %	42,2 %	37,7 %	40,2 %
Stationär*	2,4 %	3,1 %	2,8 %	3,7 %	5,4 %	4,5 %
Reha (inkl. AHB) wegen Rückenschmerzen**	4,8 %	5,4 %	5,1 %	7,2 %	9,4 %	8,2 %

* in den letzten 12 Monaten; **jemals
AHB: Anschlussheilbehandlung

Abb. 2 Rückenschmerzprävalenz und ärztliche Versorgung aufgrund von Rückenschmerzen
Quelle: Kohler M, Ziese T (2004) Telefonischer Gesundheitssurvey. Ein Wort des Robert Koch-Instituts zu chronischen Krankheiten und ihren Bedingungen. RKI, Berlin

Rückenschmerzen sind nach wie vor eine Volkskrankheit, die vor allem durch einseitige Belastungen und Bewegungsmangel entstehen. Sport und Bewegung müssen daher feste Bestandteile in der Therapie von Rückenerkrankungen sein, idealerweise auch um Rückenbeschwerden vorzubeugen.
Ein vielversprechendes Trainingskonzept ist das der sog. segmentalen Stabilisation. Unter segmentaler Stabilisation versteht man die Sicherung einzelner Bewegungssegmente der Wirbelsäule. Hauptverantwortlich sind hierfür die tiefliegenden Muskeln der Lendenwirbelsäule (z.B. Muskel M. transversus abdominis und der M. multifidus lumbalis). Es handelt sich dabei nicht um große, starke Bewegungsmuskeln, sondern um sensible „Stell- und Haltemuskeln" die einzelne Wirbelkörper in die biomechanisch richtige Position bringen, um so z.B. die Bandscheibe vor Fehlbelastungen zu schützen. In einem Review von Rackwitz (2006) (3) wurden die segmentalen Stabilisationsübungen (SSE) in Bezug auf akuten, subakuten und chronischen Rückenschmerz (low back pain) evaluiert. Die Parameter sind der Schmerz, die Behinderung durch den Rückenschmerz und die Rückkehr zum Arbeitsplatz. Der Review kommt zu folgenden Ergebnissen: Bei akutem Rückenschmerz sind SEE zur kurzfristigen Reduzierung von Schmerz und Behinderung genauso effektiv wie eine medizinische Behandlung (general practitioner). Um langfristig eine Schmerzreduzierung zu erhalten sind segmentale Stabilisationsübungen effektiver als eine medizinische Behandlung. Bei chronischem Rückenschmerz sind SEE kurz- oder langfristig gesehen effektiver als die medizinische Behandlung (general practitioner) zur Reduzierung von Schmerz und Behinderung.
Dieses Training ist allerdings noch wenig systematisch untersucht, hinsichtlich seiner Wirksamkeit auf die Kraft der Rückenmuskulatur und die Arbeitsfähigkeit von Mitarbeitern in Betrieben. In der Untersuchung sollen daher die Effekte eines angeleiteten aber selbstständig durchgeführten 20-wöchigen Trainings mit Focus auf SSE, in einer größeren Gruppe mit und ohne unspezifische lumbale Rückenbeschwerden untersucht werden.

Literatur

1. Wissenschaftliches Institut der AOK (WIdO) (2011) Die 10/20/50 Erkrankungen mit den längsten Arbeitsunfähigkeitszeiten in Tagen bei AOK-Pflichtmitgliedern ohne Rentner. WIdO, Berlin)
2. BARMER GEK (Hrsg) (2010) Gesundheitsreport 2010. Teil 1. Gesundheitskompetenz in Unternehmen stärken, Gesundheitskultur förder, www.barmer-gek.de/barmer/web/Portale/Presseportal/Subportal/Infothek/Studien und Reports/ Gesundheitsreport 2010/Teil-1-AU-Daten/Gesundheitsreport- 2010-PDF, property=Data.pdf (Stand: 24.10.2012)
3. Rackwitz B, Bie RD, Limm H, Garnier KV, Ewert T, Stucki G (2006) Segmental stabilizing exercises and low back pain. What is the evidence? A systematic review of randomized controlled trials. Clinical Rehabilitation. 20:553

3. JUSTIFICATION OF DESIGN ASPECTS

3.1 CONTROL(S) / COMPARATOR(S)

Die Interventionsgruppe wird nach den Eingangsuntersuchungen eine individuelle Anleitung zum Training der Rückenmuskulatur von den Übungsleitern erhalten. Die Übungen werden durch den Übungsleiter angelernt und durch regelmäßige Treffen (einmal pro Monat) kontrolliert und korrigiert. Die gezeigten Übungen sollen an 3 Tagen pro Woche zu je 20 min selbständig zu hause, bzw. wenn möglich auch am Arbeitsplatz, durchgeführt werden. Zusätzlich werden Mitarbeiter der AOK passende primär- uns sekundärpräventive Maßnahmen zur Rückengesundheit und zur Erhöhung der körperlichen Aktivität im Alltag heraussuchen und den Teilnehmern anbieten.

Die Kontrollgruppe wird keine Interventionen durchlaufen und gebeten ihren aktuellen Lebensstil beizubehalten. Im Anschluss an die Kontrollphase werden diese Probanden die 20-wöchige Intervention durchlaufen (Warte-Kontrollgruppe)

3.2 INCLUSION / EXCLUSION CRITERIA

<u>Inclusion criteria</u>:

- Männer und Frauen
- Alter: ≥ 18 bis 67 Jahre

<u>Exclusion criteria</u>:

- aktuelle Teilnahme an einem Programm zur Rückengesundheit
- bekannter Alkohol- oder Drogenmissbrauch
- Krankheiten (orthopädische, kardiale etc.) welche die Teilnahme an einer Trainingsintervention ausschließen
- Klinisch relevante akute oder chronische Infektionen
- schwangere Frauen

3.3 OUTCOME MEASURES

<u>Key primary endpoint</u>:

- Primärer Endpunkt ist die isometrische Rumpfkraft (Newtonmeter in 0 Grad Extension) nach 20-wöchiger Intervention gemessen als Nachher-Vorher-Differenz.

<u>Key secondary endpoints</u>:

- isometrische Messung der Kraft bei Lateralflexion
- Änderung des Rückenschmerzscores über den Oswestry Low Back Pain Disability Questionnaire (OBQ)
- Visual analog scale (VAS)
- Ermittlung der gesundheitsbezogenen Lebensqualität durch den Fragebogen SF-36
- Work-Ability-Index
- Freiburger Aktivitätsfragebogen
- Fettmasse und fettfreie Masse
- Fehl- und Krankheitstage

3.4 METHODS AGAINST BIAS

Es handelt sich um eine prospektive, kontrollierte, randomisierte und Beobachter-verblindete Studie. Es wird ein Übungsleiter die Testungen der Rückenkraft vor und nach den 20 Wochen Intervention vornehmen. Er erhält keine Information darüber, zu welcher Gruppe der Teilnehmer

randomisiert wurde. Die Trainingsvorgaben mit Instruktion werden durch einen anderen Übungsleiter vorgenommen. Die Randomisierung erfolgt an zentraler Stelle und wird nach Zentrum sowie nach dem Gesundheitsstatus der Teilnehmer (Rückenbeschwerden und keine Rückenbeschwerden) stratifiziert, da diese Variablen einen starken Einfluss auf den primären Endpunkt ausüben. Die Auswertung des primären und der wichtigsten sekundären Endpunkte erfolgt nach dem intention-to-treat-Prinzip (ITT Prinzip), um eine Überschätzung des Effekts durch drop-out zu vermeiden. Fehlende Werte werden konservativ ersetzt. Für die Rumpfkraft in Newtonmeter wird die baseline-observation-carried-forward Methode (BOCF) verwendet.

3.5 PROPOSED SAMPLE SIZE / POWER CALCULATIONS

Das primäre Ziel der Studie ist es zu zeigen, dass durch die Intervention eine Verbesserung der isometrischen Rumpfkraft nach 20 Wochen Training erreicht werden kann. Die Rumpfkraft wird in Newtonmeter gemessen, wobei hohe Werte gut sind. Betrachtet werden die Nachher-Vorher Differenzen, so dass positive Rumpfkraft-Differenzen eine Verbesserung der funktionellen Rückengesundheit bedeuten. Für die Studie wurde vorgesehen 200 Probanden einzuschließen. Daher wurde in der Planung in nQuery Advisor 7.0 berechnet, welcher Effekt bei gegebener Fallzahl gezeigt werden könnte. Die Fallzahlplanung basiert auf den Daten einer prospektiven, randomisierten Studie mit 21 Teilnehmern von Moon HJ et al., Ann Rehabil Med., 2013;37(1):110-117, in der 2 Interventionsmethoden bei Patienten mit chronischem Rückenschmerz jeweils über einen Zeitraum von 8 Wochen durchgeführt wurden. In der Gruppe mit vergleichbaren Training zu der geplanten Intervention zeigte sich eine mittlere Verbesserung von 104.0 (sd=42.5) auf 135.2 (sd=75.9). Geht man davon aus, dass sich die Kontrollgruppe nicht verändert und beide Gruppen mit den gleichen Baseline-Werten starten (Mittelwert=104.0), ergibt sich mit einem t-Test für gleiche Varianzen (größere Varianz zur konservativen Planung (sd=75.9)), einem Fehler 1. Art von 5% (zweiseitig) und einer Power von 80%, dass eine Verbesserung auf 134.22 Newtonmeter gezeigt werden kann. Dies entspricht ungefähr der Veränderung die auch bei Moon et al. gezeigt werden konnte und als relevant betrachtet wird, so dass die Fallzahl als angemessen betrachtet werden kann.

3.6 FEASIBILITY OF RECRUITMENT

Die Rekrutierung von Teilnehmer für die AOK Präventionsmaßnahme zur Rückengesundheit wird in 3 Betrieben über interne Informationsveranstaltung und betriebsinterne Werbung erfolgen. Die Mitarbeiterzahl in den Betrieben liegt bei insgesamt etwa 2000 Mitarbeitern. Aufgrund der zu erwartenden positiven Effekte der Maßnahme und der Beliebtheit eines Trainings zur Kräftigung des Bewegungsapparates erwarten wir keine Probleme bei der Rekrutierung. Wir gehen davon aus, dass die geplanten 200 Teilnehmer rekrutiert werden können.

4. STATISTICAL ANALYSIS

<u>Design</u>

Bei der geplanten Studie handelt sich um eine prospektive, randomisierte und kontrollierte Studie. Probanden aus 3 verschiedenen Betrieben, welche die Ein- und Ausschlusskriterien erfüllt haben, werden nach einer Eingangsuntersuchungen in eine Interventions- bzw. Wartelisten-Kontrollgruppe zentral 1:1 randomisiert. Die Randomisierung wird stratifiziert nach Rückenbeschwerden (ja versus nein) sowie nach Betrieb, da sie wichtige Einflussvariablen darstellen. Die Wartelisten-Kontrollgruppe erhält im Anschluss an die 20 Wochen dauernde Interventionsphase das gleiche Training.
In beiden Gruppen werden vor und nach der Intervention der primäre Endpunkt Rumpfkraft in Newtonmeter und die sekundären Endpunkte erhoben.

Primäre Auswertung:

Für die Auswertung des primären Endpunkts Rumpfkraft in Newtonmeter wird ein ANCOVA-Modell verwendet mit der mittleren Nachher-Vorher Differenz als Zielvariable. Wichtige Einflussvariablen sind die Baseline-Werte der Rumpfkraft, die Gruppen (Intervention oder Wartelisten-Kontrolle), Rückenbeschwerden (ja/nein) sowie der Betrieb. Die Intervention hat Erfolg, wenn die untere Grenze des zweiseitigen 95%-Konfidenzintervalls größer als 0 ist.

Auswertung der sekundären Endpunkte

Die wichtigsten sekundären Endpunkte werden wie der primäre Endpunkt ausgewertet. Für dichotome Endpunkte wird die logistische Regression, adjustiert für dieselben Ko-variablen, verwendet. Für alle sekundären Endpunkte werden 95%-Konfidenzintervalle berechnet.

Safety Analysis

Unerwünschte Ereignisse werden als absolute und relative Zahlen dokumentiert. Der Vergleich der Gruppen erfolgt über den Chi-quadrat Test und p-Werte werden deskriptiv betrachtet.

5. ETHICAL CONSIDERATIONS

Es bestehen keine spezifischen Risiken bei der Durchführung dieser Studie.

6 TRIAL MANAGEMENT

6.1 MAJOR PARTICIPANTS

#	Name	Affiliation	Responsibility / Role	Signature
1	Dr. Sven Haufe	Institut für Sportmedizin<br>Institut für Klinische Pharmakologie	Studienleiter	
2	Prof. Dr. Uwe Tegtbur	Institut für Sportmedizin	Stellvertretender Studienleiter	
3	Dr. Arno Kerling	Institut für Sportmedizin	Ko-investigator	
4	Prof. Dr. Armin Koch	Institut für Biometrie	Biometriker	
5	Andrea Gonnermann	Institut für Biometrie	Biometriker	
6	Lothar Stein	Institut für Sportmedizin	Ko-investigator	
7	Momme Kück	Institut für Sportmedizin	Ko-investigator	

6.2 TRIALS EXPERTISE

Das Studienteam vereint alle für das Projekt notwendigen Kompetenzen.
Dr. Sven Haufe, Sportwissenschaftler, Studienleiter und Autor zahlreicher Publikationen, Erfahrung in der Durchführung klinischer Studien und in der Betreuung von Mitarbeitern in der betrieblichen Prävention.
Prof. Dr. Uwe Tegtbur, Direktor des Instituts für Sportmedizin, Sportmediziner, hat Erfahrung in der Leitung und Durchführung von Studien zur betrieblichen Mitarbeiterprävention (u.a. Fit for Work Life als disziplinübergreifendes MHH-Präventionsangebot für Mitarbeiter, auch Kurse entsprechend dem Leitfaden Prävention sowie Rebirth-active II). Er ist Leiter des vom Deutschen Olympischen Sportbund lizenzierten Untersuchungszentrums in Niedersachsen, und erfüllt damit der Qualitätsrichtlinien für die sportmedizinische Diagnostik.

Dr. Arno Kerling, Oberarzt des Instituts für Sportmedizin, Sportmediziner. Vorerfahrung s. o.g. Studien, verantwortlicher Oberarzt für >1000 komplexe leistungsdiagnostische, sportmedizinische Untersuchungen
Lothar Stein, Sportwissenschaftler, M.A., Diplomtrainer, Vorerfahrung in der Planung und Durchführung der o.g. Studien und Projekte
H. Momme Kück; Expertise in der Studienplanung, -dokumentation und –analyse, seit 8 Jahren für o.g. wissenschaftliche Projekte.

Ausgewählte Publikationen:

Kerling A, Tegtbur U, Ziegenbein M, Grams L, Heinze DR, Sieberer M. Exercise Capacity and Quality of Life in Patients with Schizophrenia. Psychiatr Q. 2013 Mar 3. [Epub ahead of print] PubMed PMID: 23456450.

Helmer A, Kretschmer F, Deparade R, Song B, Meis M, Hein A, Marschollek M, Tegtbur U. A system for the model based emergency detection and communication for the telerehabilitation training of cardiopulmonary patients. Conf Proc IEEE Eng Med Biol Soc. ;2012:702-6. doi: 10.1109/EMBC.2012.6346028. PubMed PMID:23365989.

Dierich M, Tecklenburg A, Fuehner T, Tegtbur U, Welte T, Haverich A, Warnecke G, Gottlieb J. The influence of clinical course after lung transplantation on rehabilitation success. Transpl Int. 2013 Mar;26(3):322-30. doi: 10.1111/tri.12048. Epub 2013 Jan 7. PubMed PMID: 23294442.

Tegtbur U, Busse MW, Kubis HP. [Exercise and cellular adaptation of muscle]. Unfallchirurg. 2009 Apr;112(4):365-72. doi: 10.1007/s00113-009-1627-9. Review. German. PubMed PMID: 19308345.

Plischke M, Marschollek M, Wolf KH, Haux R, Tegtbur U. CyberMarathon - increasing physical activity using health-enabling technologies. Stud Health Technol Inform. 2008;136:449-54. PubMed PMID: 18487772.

6.3 TRIAL-SUPPORTING FACILITIES

- nicht zutreffend

7 FINANCIAL SUMMARY

**Item**	**Total funding period (€)**
Clinical project management	
Project management	
Case payment	
Data management	
Biostatistics	
Quality assurance	
Travel	
Materials	
Trial drug	
Fees, insurance	
Other	
**TOTAL**	

Die studienspezifische Arbeit wird finanziell durch die AOK gefördert.

Co-financing of the trial by a company:

Siehe oben.
